# Ribosomal S6 kinase 1 regulates inflammaging via the senescence secretome

**DOI:** 10.1038/s43587-024-00695-z

**Published:** 2024-08-29

**Authors:** Suchira Gallage, Elaine E. Irvine, Jose Efren Barragan Avila, Virinder Reen, Silvia M. A. Pedroni, Imanol Duran, Vikas Ranvir, Sanjay Khadayate, Joaquim Pombo, Sharon Brookes, Danijela Heide, Gopuraja Dharmalingham, Agharul I. Choudhury, Indrabahadur Singh, Nicolás Herranz, Santiago Vernia, Mathias Heikenwalder, Jesús Gil, Dominic J. Withers

**Affiliations:** 1 Medical Research Council Laboratory of Medical Sciences (LMS), London, UK; 2 Institute of Clinical Sciences (ICS), Faculty of Medicine, Imperial College London, London, UK; 3 Division of Chronic Inflammation and Cancer, German Cancer Research Center (DKFZ), Heidelberg, Germany; 4 University of Tübingen, Faculty of Medicine, Institute for Interdisciplinary Research on Cancer Metabolism and Chronic Inflammation, M3-Research Center for Malignome, Metabolome and Microbiome, Tübingen, Germany; 5 Emmy Noether Research Group, Division of Chronic Inflammation and Cancer, German Cancer Research Center (DKFZ), Epigenetic Machineries and Cancer, Heidelberg, Germany

**Keywords:** Senescence, Metabolic disorders, Ageing

## Abstract

Inhibition of S6 kinase 1 (S6K1) extends lifespan and improves healthspan in mice, but the underlying mechanisms are unclear. Cellular senescence is a stable growth arrest accompanied by an inflammatory senescence-associated secretory phenotype (SASP). Cellular senescence and SASP-mediated chronic inflammation contribute to age-related pathology, but the specific role of S6K1 has not been determined. Here we show that S6K1 deletion does not reduce senescence but ameliorates inflammation in aged mouse livers. Using human and mouse models of senescence, we demonstrate that reduced inflammation is a liver-intrinsic effect associated with S6K deletion. Specifically, we show that *S6K1* deletion results in reduced IRF3 activation; impaired production of cytokines, such as IL1β; and reduced immune infiltration. Using either liver-specific or myeloid-specific *S6K* knockout mice, we also demonstrate that reduced immune infiltration and clearance of senescent cells is a hepatocyte-intrinsic phenomenon. Overall, deletion of S6K reduces inflammation in the liver, suggesting that suppression of the inflammatory SASP by loss of S6K could underlie the beneficial effects of inhibiting this pathway on healthspan and lifespan.

## Abstract (web summary)

Inhibition of S6 kinase 1 (S6K1) extends lifespan in mice, but the underlying mechanisms are not fully understood. Here Gallage et al. show that reduction of S6K signaling diminishes inflammation in the aged mouse liver via suppression of the senescence-associated secretory phenotype.

## Main

The mammalian target of rapamycin (mTOR) pathway plays a key role in integrating hormone and nutrient signaling and stress responses with both cellular and organismal growth and metabolism^1^. Furthermore, mTOR signaling plays an evolutionarily conserved role in regulating longevity and healthspan^2,3^. For example, pharmacological inhibition of mTOR by rapamycin extends lifespan in yeast^4^, flies^5^ and mice^6^. A key effector of mTOR signaling is ribosomal protein S6 kinase 1 (S6K1), which plays several roles in regulating the translational machinery and controlling cellular energy levels and has feedback effects on insulin signaling^7,8^. S6K1 itself has been shown to regulate aging and different age-related processes^9,10^. Deletion of S6K1 (*Rps6kb1*) extends lifespan and healthspan in mice and also regulates longevity in flies and worms^9,11^.

Mice lacking S6K1 display beneficial metabolic effects, including reduced adipose mass, resistance to the consequences of high-fat diet feeding and increased insulin sensitivity^12,13^, a constellation of phenotypes that aligns with the effects of calorie restriction (CR), a conserved longevity mechanism^14^. Different molecular mechanisms have been proposed to explain these effects. For example, loss of S6K1 leads to upregulation of the activity of AMP kinase, a key regulator of cellular energy homeostasis^15^, thus mimicking the effects of CR and motivating the use of metformin as a potential geroprotective drug^16^. S6K1 also phosphorylates the glutamyl-prolyl-tRNA synthetase (EPRS), which, in turn, is involved in regulating adiposity and adipose tissue metabolism, and this may underlie the beneficial metabolic phenotypes observed in S6K1 null mice^17^. Despite these insights, a definitive answer to what are the cellular and molecular mechanisms behind the broad-ranging effects associated with the abrogation of S6K signaling is unclear.

Cellular senescence is a stress response that limits the replication of old, damaged and cancerous cells^18^. Senescent cells stably exit the cell cycle and undergo multiple phenotypic changes, including the production of a pro-inflammatory secretome known as the senescence-associated secretory phenotype (SASP)^19^. Cellular senescence is a hallmark of aging^20^: senescent cells not only accumulate during aging^21^ but also contribute to aging and age-related diseases^22^.

Interestingly, mTOR influences different phenotypes associated with senescence^14^. Inhibition of mTOR prevents senescence by interfering with the establishment of an irreversible growth arrest^23,24^. On the other hand, treatment of already senescent cells with rapamycin inhibits the inflammatory SASP^25,26^. Mechanistically, 4EBP has been implicated in mTOR-mediated SASP regulation^25,26^, but the role of S6K signaling has not been investigated. Moreover, the S6K–STING interaction has been shown to regulate immune responses^27^. Because the cGAS/STING pathway is central to regulating the SASP^28,29^, it is tempting to hypothesize whether S6K could regulate the inflammatory SASP.

Given our incomplete understanding of the mechanisms by which loss of S6K1 signaling benefits aging and age-related pathologies, we undertook a series of studies in long-lived *S6K1*^*−/−*^ mice and other genetic and pharmacological models of S6K inhibition, including liver-specific and myeloid-specific S6K knockout (KO) mice. We explored the role of senescence, the SASP and inflammation in the liver, as this organ displays several age-related changes (including an increased inflammatory profile^30,31^) and shows beneficial metabolic phenotypes in mice lacking S6K1 (refs. ^32,33^). In these studies, we found that loss of S6K1 attenuates age-related liver pathology and does not influence senescence but reduces liver inflammation via effects on the pro-inflammatory SASP and immune surveillance. Thus, S6K signaling plays a key role in age-related inflammation (inflammaging^34^), and targeting this pathway may be a strategy for treating the diseases of aging.

## Results

### *S6K1* deletion attenuates age-related liver pathology

**Fig. 1:**
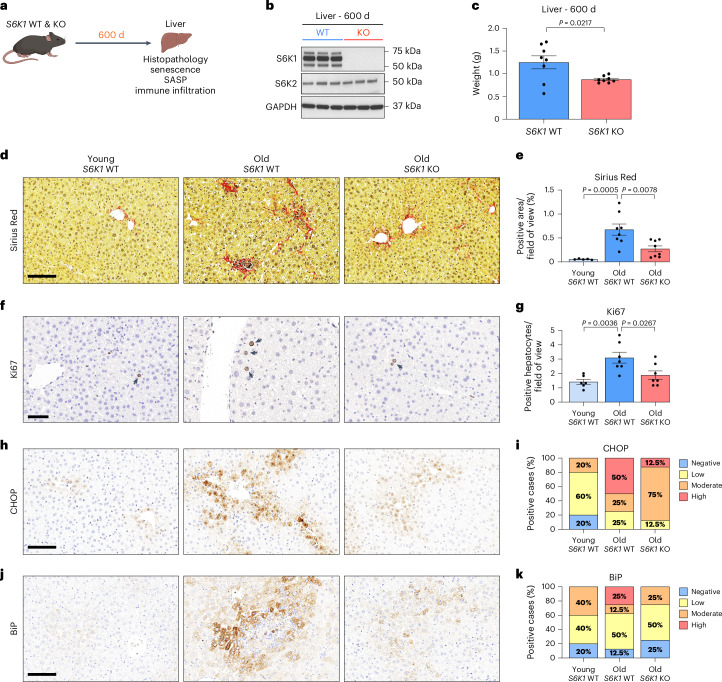
S6K1 deletion attenuates age-related liver pathology. **a**, Experimental scheme. *S6K1* WT and KO mice were aged for 600 d to assess senescence. **b**, Immunoblot images of S6K1, S6K2 and GAPDH protein expression in whole liver lysates of 600-day-old *S6K1* WT (left; *n* = 3) and KO (right; *n* = 3) mice. GAPDH acted as a loading control for S6K1. S6K2 was run on a separate blot (and, therefore, GAPDH is a sample preparation control for that blot). **c**, Liver weight (grams) at 600 d from *S6K1* WT (*n* = 8) and KO (*n* = 8) mice. **d**,**e**, Sirius Red staining (**d**) and quantification (**e**) in livers in young *S6K1* WT (90 d; *n* = 5), old *S6K1* WT (600 d; *n* = 8) and old *S6K1* KO (600 d; *n* = 8) mice. **f**,**g**, Ki67 staining (**f**) and quantification (**g**) in livers in young *S6K1* WT (90 d; *n* = 6), old *S6K1* WT (600 d; *n* = 7) and old *S6K1* KO (600 d; *n* = 7) mice. **h**,**i**, CHOP staining (**h**) and quantification (**i**) in livers in young *S6K1* WT (90 d; *n* = 5), old *S6K1* WT (600 d; *n* = 8) and old *S6K1* KO (600 d; *n* = 8) mice. **j**,**k**, BiP staining (**j**) and quantification (**k**) in livers in young *S6K1* WT (90 d; *n* = 5), old *S6K1* WT (600 d; *n* = 8) and old *S6K1* KO (600 d; *n* = 8) mice. Data are expressed as mean ± s.e.m. Statistical significance was calculated using either a two-tailed Student’s *t*-test (**c**) or one-way ANOVA with Tukey’s multiple comparison test (**e**,**g**). *n* denotes individual mice. Scale bar, 100 μm (**d**,**h**,**j**) or 50 μm (**f**). Source data

**Extended Data Fig. 1:**
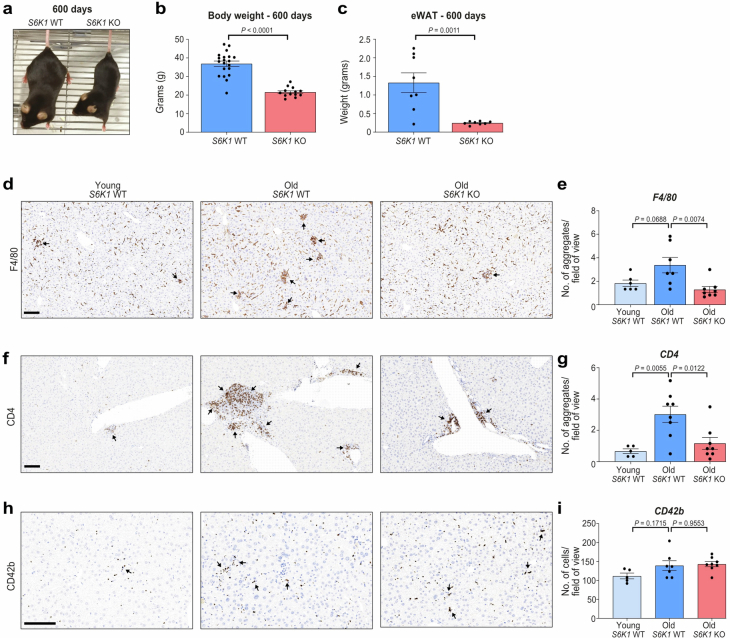
S6K1 deletion attenuated age-induced obesity and ‘inflammaging’ in the liver. *S6K1* wild-type (WT) and knockout (KO) mice were aged for 600 days. **a**. Representative photograph of 600-day-old *S6K1* WT (left) and KO (right) mice. **b**. Body weight (grams) at 600 days of *S6K1* WT (left; n = 20) and KO (right; n = 13) mice. **c**. Epididymal white adipose tissue (eWAT) weight (grams) at 600 days of *S6K1* WT (left; n = 8) and KO (right; n = 8) mice. **d-e**. F4/80 staining for resident Kupffer cells **(d)** and quantification **(e)** of livers from young *S6K1* WT (90 days; n = 6), old *S6K1* WT (600 days; n = 7) and old *S6K1* KO (600 days; n = 8) mice. **f-g**. CD4 staining for T-helper cells **(f)** and quantification **(g)** of livers in young *S6K1* WT (90 days; n = 5), old *S6K1* WT (600 days; n = 8) and old *S6K1* KO (600 days; n = 8) mice. **h-i** CD42b staining for platelets **(h)** and quantification **(i)** of livers in young *S6K1* WT (90 days; n = 5), old *S6K1* WT (600 days; n = 7) and old *S6K1* KO (600 days; n = 8) mice. Data are expressed as mean ± SEM. Statistical significance was calculated using either a two-tailed Student’s t-test (**b-c**) or a one-way analysis of variance with Tukey’s multiple comparison test (**e**, **g**, **i**). *n* denotes individual mice. Scale bar, 100 μm. Data are expressed as mean ± SEM. Statistical significance was calculated using. *n* denotes individual mice. Source data

To investigate the role of S6K1 in age-related liver pathology, particularly in the regulation of senescence and the SASP, we compared the livers of old (600 d) *S6K1* wild-type (WT) and KO female mice (Fig. 1a,b). To this end, we established two cohorts of mice. As previously described, *S6K1* KO mice were smaller than age-matched *S6K1* WT littermates (Extended Data Fig. 1a,b) and displayed significantly reduced liver and epididymal white adipose tissue (eWAT) mass (Fig. 1c and Extended Data Fig. 1c).

Senescent cells contribute to age-related liver pathology, including hepatic steatosis^35^ and inflammation^31^ as well as liver fibrosis^36,37^. Consistent with previous evidence of preserved organ homeostasis and function in old age, *S6K1* KO mice showed improved liver pathology (Fig. 1d–k). Sirius Red staining of liver sections showed that old *S6K1* WT mice displayed increased levels of fibrosis as compared to their younger counterparts, whereas fibrosis was significantly lower in old *S6K1* KO littermates (Fig. 1d,e). An increase in the numbers of Ki67^+^ hepatocytes observed in the livers of old *S6K1* WT mice, indicative of compensatory proliferation in response to age-related liver damage, was reduced in the livers of old *S6K1* KO littermates (Fig. 1f,g). Markers of endoplasmic reticulum (ER) stress, such as CHOP and BiP, that reflect liver damage, were also found elevated in the livers of old *S6K1* WT mice, whereas this was attenuated in the livers of old *S6K1* KO littermates (Fig. 1h–k). Overall, these data confirm that old *S6K1* KO mice display increased liver fitness as reflected by amelioration of age-related liver pathology.

### S6K1 status affects inflammation but not senescence in the livers of old mice

**Fig. 2:**
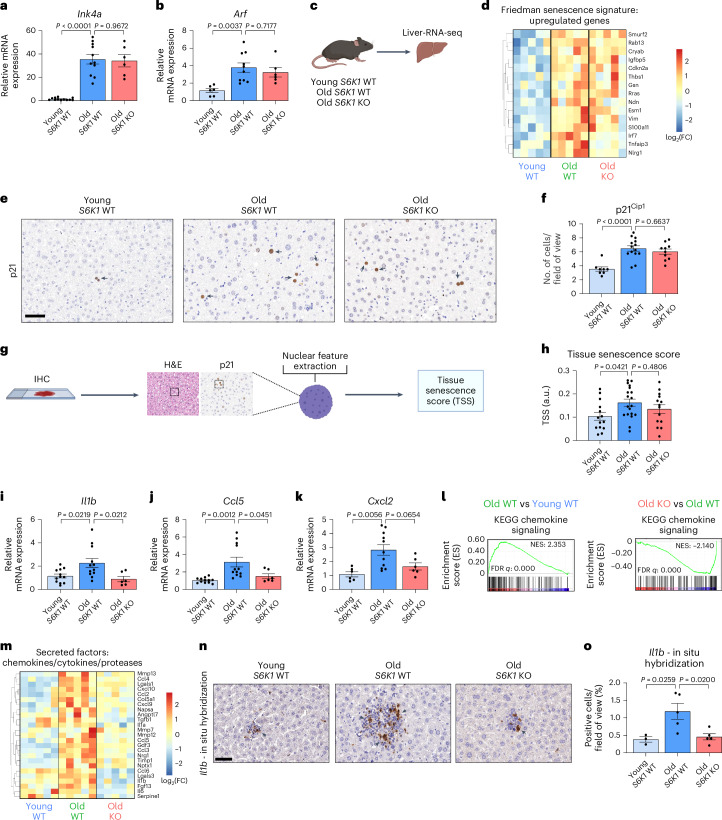
S6K1 status affects inflammation but not senescence in the livers of old mice. **a**,**b**, Relative mRNA expression for *Ink4a* (**a**) and *Arf* (**b**) were assessed by RT–qPCR from whole liver lysates of young *S6K1* WT (90 d; *n* = 12 *Ink4a* and *n* = 6 *Arf*), old *S6K1* WT (600 d; *n* = 11 *Ink4a* and *n* = 10 *Arf*) and old *S6K1* KO (600 d; *n* = 6 for *Ink4a* and *Arf*) mice. mRNA expression was normalized to the *Rps14* housekeeping gene. **c**, Experimental scheme. Bulk RNA-seq of whole liver lysates from young *S6K1* WT (90 d), old *S6K1* WT (600 d) and old *S6K1* KO (600 d) mice. **d**, Heatmap depicting the expression of key regulated genes in the ‘Friedman senescence signature’ in young *S6K1* WT (90 d; *n* = 5), old *S6K1* WT (600 d; *n* = 5) and old *S6K1* KO (600 d; *n* = 5) mice. **e**,**f**, p21 staining (**e**) and quantification (**f**) of young *S6K1* WT (90 d; *n* = 8), old *S6K1* WT (600 d; *n* = 14) and old *S6K1* KO (600 d; *n* = 10) mice. Scale bar, 50 µm. **g**, Pipeline for calculating TSSs based on nuclear parameter extraction from H&E-stained liver tissue slides. **h**, TSSs of young S6K1 WT (90 d, *n* = 14), old S6K1 WT (600 d, *n* = 19) and old S6K1 KO (600 d, *n* = 14) mice. **i**–**k**, Relative mRNA expression for *Il1b* (**i**)*, Ccl5* (**j**) and *Cxcl2* (**k**) assessed by RT–qPCR from whole liver lysates of young *S6K1* WT (90 d; *n* = 12 for *Il1b* and *Ccl5*; *n* = 6 for *Cxcl2*), old *S6K1* WT (60 d; *n* = 12 for *Il1b* and *Ccl5*; *n* = 11 for *Cxcl2*) and old *S6K1* KO (60 d; *n* = 6 for *Il1b*, *Ccl5* and *Cxcl2*) mice. mRNA expression was normalized to the *Rps14* housekeeping gene. **l**, GSEA for ‘KEGG Chemokine Signaling’ of young *S6K1* WT (90 d), old *S6K1* WT (600 d) and old *S6K1* KO (600 d) mice from whole liver lysates. **m**, Heatmap depicting the expression of key chemokines, cytokines and proteases in young *S6K1* WT (90 d; *n* = 5), old *S6K1* WT (600 d; *n* = 5) and old *S6K1* KO (600 d; *n* = 5) mice. **n**,**o**, In situ hybridization for *Il1b* mRNA (**n**) and quantification (**o**) in young *S6K1* WT (90 d; *n* = 3), old *S6K1* WT (600 d; *n* = 5) and old *S6K1* KO (600 d; *n* = 5) mice. Scale bar, 50 µm. Data are expressed as mean ± s.e.m. Statistical significance was calculated using one-way ANOVA with Tukey’s multiple comparison test. *n* denotes individual mice. FC, fold change; FDR, false discovery rate; IHC, immunohistochemistry; NES, normalized enrichment score. Source data

Senescent cells accumulate during aging^21^ and contribute to age-related tissue dysfunction through the production of the SASP^38^. We hypothesized that regulation of senescence by S6K1 may explain, at least in part, the beneficial effects that *S6K1* loss has on age-related liver pathology. However, markers of senescence, such as the *Cdkn2a* transcripts encoding for p16^Ink4a^ (*Ink4a*; Fig. 2a) and p19^Arf^ (*Arf*; Fig. 2b), were upregulated in old mice irrespective of the genotype, suggesting that S6K1 status did not affect the senescence response per se. Moreover, gene set enrichment analysis (GSEA) performed on RNA sequencing (RNA-seq) of liver samples (Fig. 2c) unveiled senescence-related signatures that were upregulated in old animals, irrespective of S6K1 status (Fig. 2d). Likewise, we detected no difference in p21^Cip1^ staining in the livers of old S6K1 KO mice compared to aged-matched WT animals (Fig. 2e,f). We also took advantage of a machine learning algorithm that relies on the characteristic nuclear features of senescent cells to identify senescence in hematoxylin and eosin (H&E)-stained tissue sections^39^ (Fig. 2g). The tissue senescence scores (TSSs) calculated using this approach showed no significant senescence attenuation in the livers of old S6K1 KO mice compared to age-matched control animals (Fig. 2h). Taken together, these data suggest that S6K1 loss did not affect senescence induction.

Senescent cells produce a complex mix of immunomodulatory cytokines referred to as the SASP^19^. The expression of several immunomodulatory cytokines in the liver, including *Il1b*, *Ccl5* and *Cxcl2* (Fig. 2i–k), were elevated during aging in *S6K1* WT mice but were downregulated in old *S6K1* KO animals. Furthermore, GSEA showed that a chemokine signaling signature was elevated in old *S6K1* WT mice when compared to young control animals, and this was also downregulated in old *S6K1* KO littermates (Fig. 2l). Heatmaps confirmed that the expression of multiple cytokines increased with age in the livers of *S6K1* WT mice while remaining at lower levels in *S6K1* KO mice (Fig. 2m). Finally, RNA in situ hybridization of liver sections showed that *Il1b* was elevated in livers of old *S6K1* WT mice when compared to livers of old *S6K1* KO mice (Fig. 2n,o). These results suggest that *S6K1* loss does not affect cellular senescence but, rather, impairs the production of a subset of inflammatory cytokines during aging.

### *S6K1* deletion prevents inflammaging in liver

**Fig. 3:**
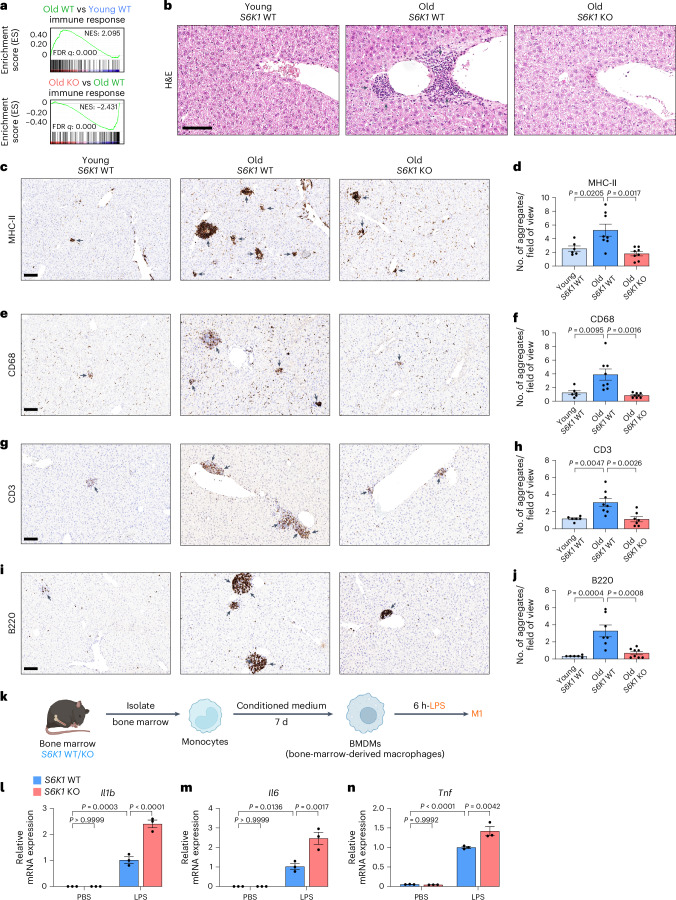
S6K1 deletion prevents inflammaging in livers. **a**, GSEA for ‘Immune Response’ of young *S6K1* WT (90 d), old *S6K1* WT (600 d) and old *S6K1* KO (600 d) mice from whole liver lysates. **b**, H&E staining of livers from mice of the indicated genotypes. **c**,**d**, MHC-II staining for antigen-presenting cells (**c**) and quantification (**d**) of livers from young *S6K1* WT (90 d; *n* = 6), old *S6K1* WT (600 d; *n* = 8) and old *S6K1* KO (600 d; *n* = 8) mice. **e**,**f**, CD68 staining for monocytes and macrophages (**e**) and quantification (**f**) of livers from young *S6K1* WT (90 d; *n* = 6), old *S6K1* WT (600 d; *n* = 8) and old *S6K1* KO (600 d; *n* = 8) mice. **g**,**h**, CD3 staining for T cells (**g**) and quantification (**h**) of livers from young *S6K1* WT (90 d; *n* = 6), old *S6K1* WT (600 d; *n* = 8) and old *S6K1* KO (600 d; *n* = 7) mice. **i**,**j**, B220 staining for B cells (**i**) and quantification (**j**) of livers from young *S6K1* WT (90 d; *n* = 6), old *S6K1* WT (600 d; *n* = 7) and old *S6K1* KO (600 d; *n* = 8) mice. **k**, Experimental scheme for stimulation of BMDMs. BMDMs were generated from *S6K1* WT/KO mice and treated with 100 ng ml^−1^ LPS for 6 h. **l**–**n**, Relative mRNA expression of *Il1b* (**l**)*, Il6* (**m**) and *Tnfa* (**n**) assessed by RT–qPCR. mRNA expression was normalized to the *Rps14* housekeeping gene (*n* = 4). Data are expressed as mean ± s.e.m. Statistical significance was calculated using one-way ANOVA with Tukey’s multiple comparison test. *n* denotes individual mice. Scale bar, 100 μm. FDR, false discovery rate; NES, normalized enrichment score. Source data

**Extended Data Fig. 2:**
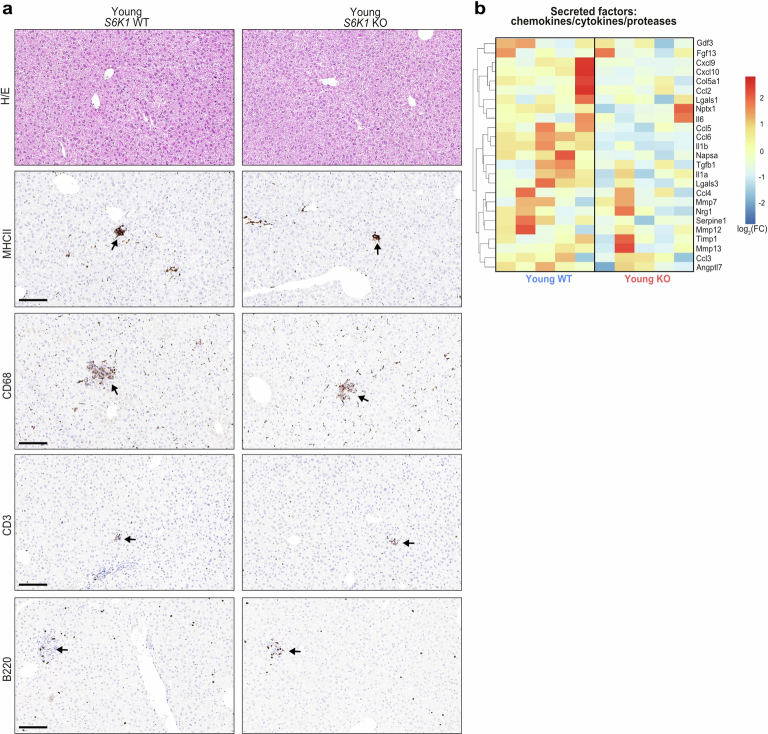
S6K1 deletion does not affect immune infiltration in young mice. Immunohistochemistry analysis of immune cell markers in young (90 days) *S6K1* WT and KO mice. **a**. Haematoxylin and eosin (H/E) staining, MHCII staining for antigen-presenting cells, CD68 staining for monocytes and macrophages, CD3 staining for T cells and B220 staining for B cells in liver sections. Scale bar, 100 μm. **b**. Heatmap depicting the expression of key chemokines, cytokines and proteases in young *S6K1* WT (90 days; n = 5) and young *S6K1* KO (90 days; n = 5) mice. WT, wild-type. KO, knockout. Data are representative of a single experiment.

**Extended Data Fig. 3:**
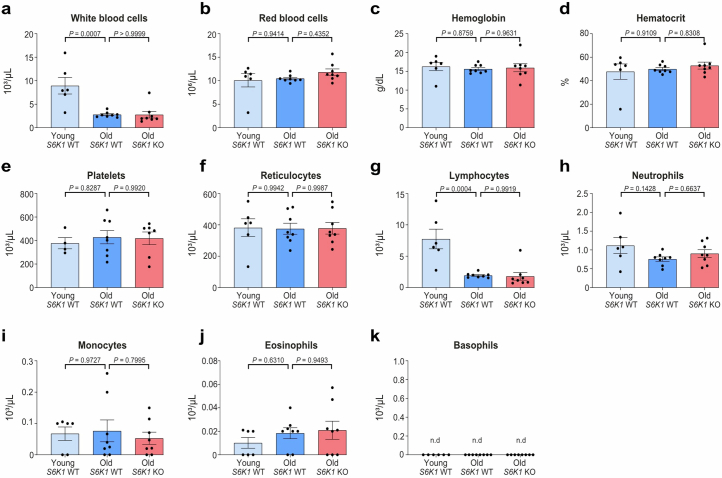
S6K1 deletion does not significantly alter the systemic blood count during ageing. Whole blood of young *S6K1* WT (90 days; n = 6 for all measures except n = 4 for platelets), old *S6K1* WT (600 days; n = 8) and old *S6K1* KO (600 days; n = 8) mice were used to assess the full blood count. **a**. White blood cell count (10^3^/μL). **b**. Red blood cell count (10^6^/μL). **c**. Haemoglobin (g/dL). **d**. Haematocrit (%). **e**. Platelet count (10^3^/μL). **f**. Reticulocytes (10^3^/μL). **g**. Lymphocytes (10^3^/μL). **h**. Neutrophils (10^3^/μL). **i**. Monocytes (10^3^/μL). **j**. Eosinophils (10^3^/μL). **k**. Basophils (10^3^/μL). Data are expressed as mean ± SEM. Statistical significance was calculated using a one-way analysis of variance with Tukey’s multiple comparison test. *n* denotes individual mice. Source data

The accumulation of senescent cells has been associated with chronic inflammation and persistent immune cell infiltrates during aging, often referred to as inflammaging^34,38^. Given that the livers of old *S6K1* WT mice displayed lower expression of inflammatory, immunomodulatory cytokines, we speculated that this might result in decreased levels of chronic immune infiltration in old mice. GSEA showed that an immune response signature was significantly upregulated in the livers of old *S6K1* WT mice when compared to either young mice or old *S6K1* KO mice (Fig. 3a). Moreover, H&E staining of liver sections showed increased immune infiltration in old *S6K1* WT mice that was less pronounced in age-matched *S6K1* KO littermates (Fig. 3b). To study this in more detail, we used quantitative immunohistochemistry on whole liver sections for various immune cell markers. We previously observed an increase in myeloid and lymphoid infiltrates in the liver of aged mice^31^. In agreement with those results, an increase in myeloid (major histocompatibility complex II (MHC-II^+^, CD68^+^ and F4/80^+^)) and lymphoid (CD3^+^ and B220^+^) infiltrates was observed in old *S6K1* WT mice (Fig. 3c–j and Extended Data Fig. 1d–i). Interestingly, age-matched *S6K1* KO littermates display reduced infiltration of myeloid cells (MHC-II^+^, CD68^+^ and F4/80^+^ cells; Fig. 3c–f and Extended Data Fig. 1d,e), T cells (CD3^+^ or CD4^+^ cells; Fig. 3g,h and Extended Data Fig. 1f,g) and B cells (B220^+^ cells; Fig. 3i,j). We did not observe significant differences in the presence of platelet infiltration (Extended Data Fig. 1h,i), suggesting that the effect was specific. There were no striking differences in immune infiltrates in the livers of young *S6K1* WT and KO mice (Extended Data Fig. 2a). Chemokine and cytokine expression in the liver of young WT and S6K1 KO mice showed that basal levels were reduced in the younger KO animals (Extended Data Fig. 2b), suggesting reduced basal expression of inflammatory factors upon S6K1 deletion. Moreover, no differences were observed in the levels of circulating monocytes/lymphocytes in the blood taken from the respective cohorts, suggesting that the observed changes were due to differences in infiltration (Extended Data Fig. 3).

The reduced infiltration of immune cells in the livers of S6K1 KO mice may result from potentially cell-intrinsic alterations in immune cell function associated with the global loss of S6K1. To address this issue, we studied bone-marrow-derived macrophages (BMDMs) isolated from S6K1 KO mice and control animals. We observed no deficiency in response to lipopolysaccharide (LPS) as measured by mRNA levels of *Il1b*, *Il6* and *Tnf*, indicating that loss of S6K1 in BMDMs did not impair immune activation (Fig. 3k–n).

Taken together, these results suggest that the lower infiltration of specific immune cell types observed in the livers of old *S6K1* KO mice was due to their specific recruitment into the liver, rather than an alteration in populations or numbers in peripheral blood, and that reduced inflammation was not due to intrinsic changes in immune cell activation caused by loss of S6K1. Overall, the above results suggest that, although S6K1 loss does not result in reduced numbers of senescent cells in the liver of old WT mice, the livers of old *S6K1* KO mice showed less chronic inflammation and immune infiltration than old *S6K1* WT controls.

### S6K regulates the inflammatory SASP in mouse embryonic fibroblasts

**Fig. 4:**
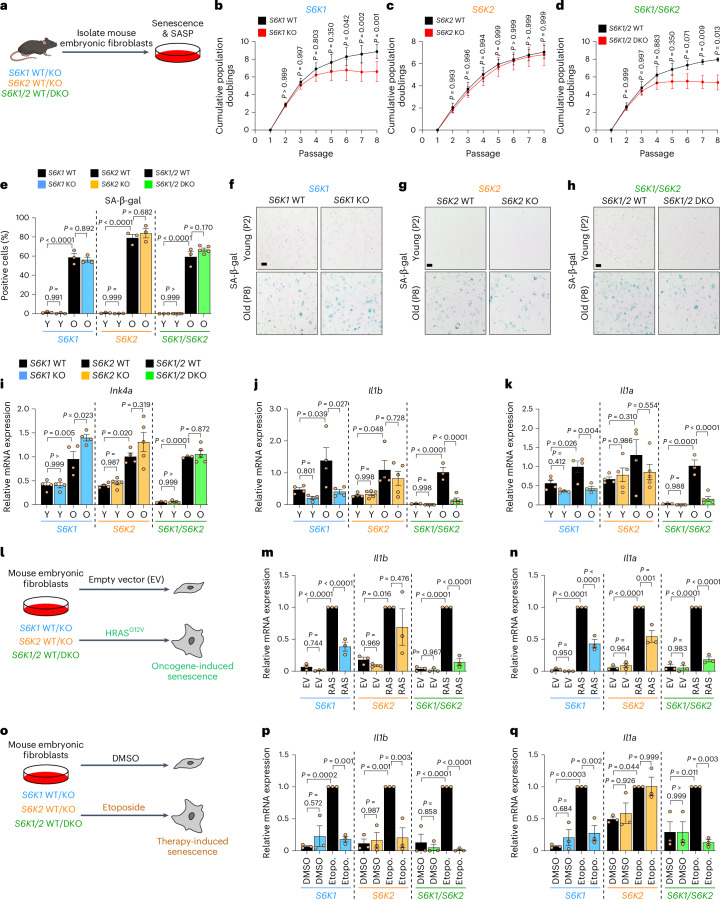
S6K1 and/or S6K2 deletion does not bypass senescence but dampens SASP induction in MEFs. **a**, Experimental scheme. MEFs were generated from *S6K1* WT/KO, *S6K2* WT/KO and *S6K1/2* WT/DKO embryos and were assessed for replicative senescence. MEFs were generated from 3­-5 independent pairs of embryos from at least three different mothers. **b**–**d**, Cumulative population doublings of *S6K1* WT (*n* = 5) and KO (*n* = 4) MEFs (**b**), *S6K2* WT (*n* = 4) and KO (*n* = 5) MEFs (**c**) and *S6K1/2* WT (*n* = 3) and DKO (*n* = 5) MEFs (**d**). **e**–**h**, Quantification (**e**) and representative images (**f**–**h**) of SA-β-gal staining in young (passage 2) and old (passage 8) MEFs from *S6K1* WT and KO (young *n* = 3; old *n* = 3), *S6K2* WT and KO (young *n* = 3; old *n* = 3) and *S6K1/2* WT (young *n* = 3; old *n* = 3) and DKO (young *n* = 4; old *n* = 5) cells. Scale bar, 100 μm. **i**–**k**, Relative mRNA expression for *Ink4a* (**i**), *Il1b* (**j**) and *Il1a* (**k**) assessed by RT–qPCR from young (passage 3) and old (passage 8) MEFs from *S6K1* WT (*n* = 4) and KO (*n* = 4), *S6K2* WT (*n* = 4) and KO (*n* = 5) as well as *S6K1/2* WT (*n* = 3) and DKO (*n* = 5) cells. mRNA expression was normalized to the *Rps14* housekeeping gene. **l**, Experimental scheme. MEFs of the indicated genotypes were stably transduced with a retroviral vector containing the EV or expressing HRAS^G12V^. MEFs were generated from three independent pairs of embryos from three different mothers. **m**,**n**, Relative mRNA expression for *Il1b* (**m**) and *Il1a* (**n**) assessed by RT–qPCR from MEFs transduced with EV or *HRAS*^*G12V*^ (RAS) from *S6K1* WT (*n* = 3) and KO (*n* = 3), *S6K2* WT (*n* = 3) and KO (*n* = 3) as well as *S6K1/2* WT (*n* = 3) and DKO (*n* = 3) cells. mRNA expression was normalized to the *Rps14* housekeeping gene. **o**, Experimental scheme. MEFs of the indicated genotypes were treated with DMSO or 5 μM etoposide for 7 d. MEFs were generated from three independent pairs of embryos from three different mothers. **p**,**q**, Relative mRNA expression for *Il1b* (**p**) and *Il1a* (**q**) assessed by RT**–**qPCR from MEFs treated with DMSO or 5 μM etoposide from *S6K1* WT (*n* = 3) and KO (*n* = 3), *S6K2* WT (*n* = 3) and KO (*n* = 3) as well as *S6K1/2* WT (*n* = 3) and DKO (*n* = 3) cells. mRNA expression was normalized to the *Rps14* housekeeping gene. Data are expressed as mean ± s.e.m. Statistical significance was calculated using repeated two-way ANOVA with Sidak’s multiple comparison test (**b–d**) or by two-way ANOVA with Tukey’s multiple comparison test (**e**,**i**–**k**,**m**,**n**,**p**,**q**). *n* denotes individual MEF replicates derived from different embryos. Etopo., etoposide; O, old; P, passage; Y, young. Source data

A possible explanation for the phenotypes observed in the livers of *S6K1* KO mice is that *S6K1* loss results in selective inhibition of pro-inflammatory cytokines without affecting other senescence phenotypes. To investigate this possibility, we took advantage of mouse embryonic fibroblasts (MEFs) derived from *S6K1* KO, *S6K2* KO or *S6K1/S6K2* double knockout (DKO) mice and compared them to their WT counterparts (Fig. 4a).

**Extended Data Fig. 4:**
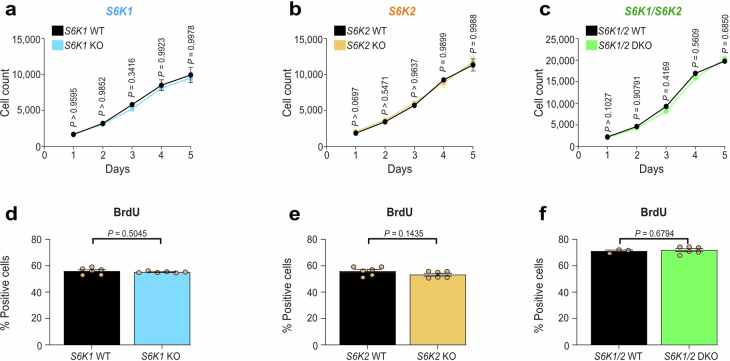
S6K1 and/or S6K2 deletion do not affect proliferation at early passage in mouse embryonic fibroblasts. Mouse embryonic fibroblasts (MEFs) from an early passage (passage 2) *S6K1* WT/KO, *S6K2* WT/KO and *S6K1/2* WT/DKO embryos were assessed for cell count and proliferation of the indicated genotypes. **a-c**. The time course of cell count was assessed by high throughput microscopy of DAPI staining for 5 days. **d-f**. Percentage of BrdU-positive cells on day 3 of the indicated genotypes. Data are expressed as mean ± SEM. Statistical significance was calculated using either a two-way analysis of variance with Šidák’s multiple comparison test (**a-c**) or a two-tailed Student’s t-test (**d-f**). WT, wild-type. KO, knockout. DKO, double knockout. n = 6 biological replicates from a single experiment (**a-b** and **d-e**). n = 3 (WT) and n = 6 (DKO) biological replicates from a single experiment (**c** and **f**). MEFs isolated from 2 independent pairs of embryos. n = 3-6 biological replicates from a single experiment (**c** and **f**). Source data

**Extended Data Fig. 5:**
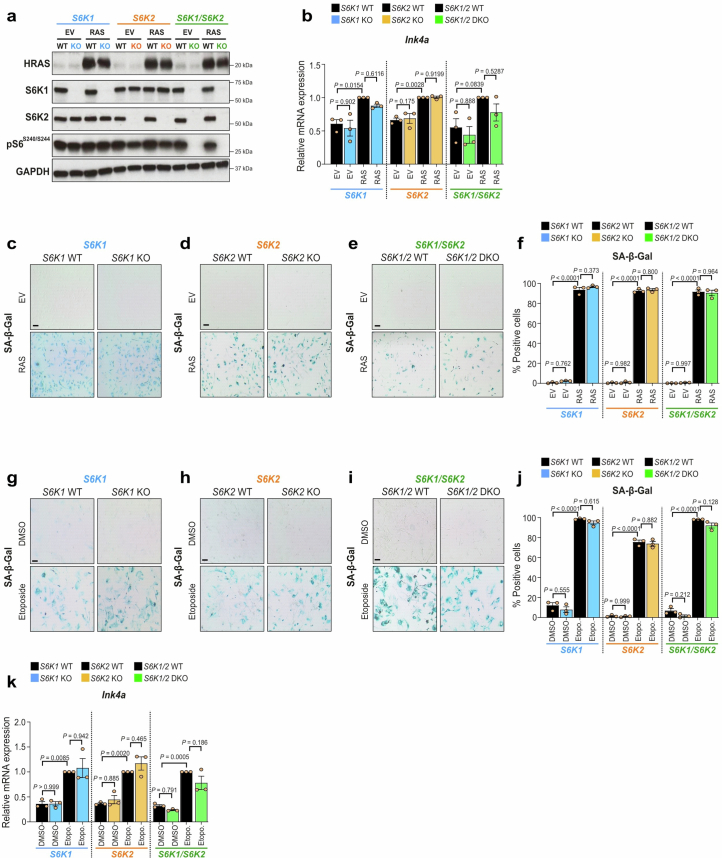
Induction of senescence in MEFs with different S6K1 and S6K2 status. **a**. Immunoblot images of a single experiment for HRAS, S6K1, S6K2, pS6^S240/S244^ and GAPDH expression. S6K1 and GAPDH (loading control) were run on the same blot. S6K2, pS6^S240/S244^ and HRAS were run on separate blots; therefore, GAPDH served as a sample preparation control for those blots. **b**. Relative mRNA expression for *Ink4a* assessed by RT-qPCR from MEFs transduced with empty vector (EV) or *HRAS*^*G12V*^ (RAS) from *S6K1* WT (n = 3) and KO (n = 3), *S6K2* WT (n = 3) and KO (n = 3) as well as *S6K1/2* WT (n = 3) and DKO (n = 3) cells. **c-f**. Quantification **(f)** and representative images **(c-e)** of senescence-associated beta-galactosidase (SA-β-Gal) staining in S6K1, S6K2, and S6K1/2 MEFs with the indicated genotype expressing either EV or RAS vector to undergo OIS (n = 3*)*. **g-j**. Quantification **(j)** and representative images **(g-i)** of senescence-associated beta-galactosidase (SA-β-Gal) staining in S6K1, S6K2, and S6K1/2 MEFs with the indicated genotype treated with DMSO or etoposide (5 μM) to undergo TIS (n = 3). **k**. Relative mRNA expression for *Ink4a* was assessed by RT-qPCR from MEFs treated with DMSO or etoposide (5 μM) to undergo TIS (n = 3). mRNA expression was normalized to the *Rps14* housekeeping gene. Data are expressed as mean ± SEM. Statistical significance was calculated using two-way analysis of variance with Tukey’s multiple comparison test. *n* denotes individual MEFs generated from independent embryos. WT, wild-type. KO, knockout. DKO, double knockout. Scale bar, 100 μm. Source data

Loss of *S6K1* and/or *S6K2* did not abrogate the growth arrest observed during serial passages of MEFs. Indeed, *S6K1* KO and *S6K1/S6K2* DKO cells were arrested even earlier than WT MEFs (Fig. 4b–d). This premature arrest, observed in *S6K1* KO and *S6K1/S6K2* DKO MEFs subjected to serial passage, was not due to intrinsic differences in cell growth (Extended Data Fig. 4a–f). Late-passage *S6K1* KO, *S6K*2 KO and *S6K1/S6K2* DKO MEFs showed similar senescence-associated β-galactosidase (SA-β-gal) cell staining to their WT counterparts (Fig. 4e–h). Further analysis confirmed that *Ink4a*, the transcript encoding for p16^Ink4a^, a CDKI necessary for senescence growth arrest, was similarly induced in old MEFs regardless of *S6K1* and *S6K2* deletion (Fig. 4i). Nevertheless, the induction of SASP components, such as the pro-inflammatory cytokines *Il1a* and *Il1b* observed in late-passage MEFs, was significantly reduced in *S6K1* KO and *S6K1/S6K2* DKO but not S6K2 KO MEFs when compared to WT cells (Fig. 4j,k). We next analyzed whether these effects were also observed during oncogene-induced senescence (OIS). We, therefore, infected MEFs with an oncogenic *Ras* (HRAS^G12V^)-expressing vector or its parental vector as a control (Fig. 4l and Extended Data Fig. 5a). *Ras* expression triggered senescence induction regardless of *S6K1* and *S6K2* status, as shown by analysis of *Ink4a* transcript levels (Extended Data Fig. 5b) or SA-β-gal staining (Extended Data Fig. 5c–f). Staining for SA-β-gal activity showed that there was no reduction in OIS in *S6K1* KO, *S6K*2 KO and *S6K1/S6K2* DKO MEFs compared to control cells (Extended Data Fig. 5c–f). In contrast, the induction of *Il1b* (Fig. 4m) and *Il1a* (Fig. 4n) observed during OIS was significantly reduced in *S6K1* KO, *S6K*2 KO and *S6K1/S6K2* DKO MEFs when compared to WT cells. Finally, we also studied the effects of therapy-induced senescence (TIS) using etoposide treatment of WT and *S6K1* and/or *S6K2* KO MEFs (Fig. 4o). We found equivalent senescence induction in *S6K1* KO, *S6K*2 KO and *S6K1/S6K2* DKO MEFs compared to WT cells, as judged by SA-β-gal cell staining (Extended Data Fig. 5g–j) and expression of *Ink4a*, the transcript encoding for p16^Ink4a^ (Extended Data Fig. 5k). In contrast, *S6K1* KO and *S6K1/S6K2* DKO MEFs showed reduced expression of *Il1b* and *Il1a* in response to etoposide treatment (Fig. 4p–q). The above results suggest that deletion of *S6Ks* results in impaired production of pro-inflammatory cytokines during senescence, with combined loss of *S6K1* and *S6K2* having the strongest effect.

### S6K1/2 regulates the pro-inflammatory SASP in human cells

**Fig. 5:**
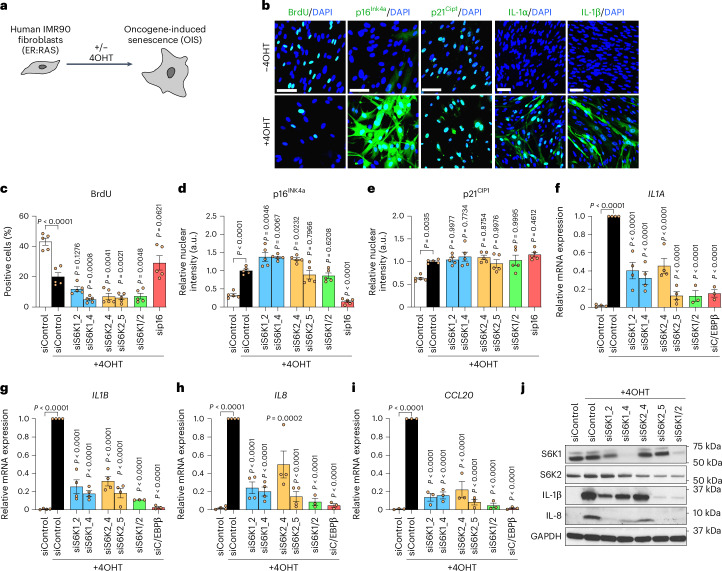
S6K1/2 regulates the SASP without affecting the growth arrest in human fibroblasts undergoing OIS. **a**, Experimental scheme. IMR90 fibroblasts were stably transduced with the pLNC-ER:RAS retroviral vector and treated with 4OHT for senescence induction. **b**, Representative IF staining of BrdU, p16^INK4A^, IL-1α, IL-1β and IL-8 after 7 d (BrdU and p16^Ink4a^) or 8 d (SASP) with or without 4OHT treatment in IMR90 ER:RAS cells. Scale bar, 100 μm. **c**–**e**, IMR-90 ER:RAS cells were reverse transfected with either AllStars (scrambled sequence, siControl) or the indicated siRNAs. Cells were treated with or without 4OHT on the following day to induce senescence. Quantification of IF staining for BrdU incorporation (**c**), p16^INK4A^ (**d**) and p21^CIP1^ (**e**) after 5 d of 4OHT treatment (*n* = 5 biological replicates from two independent experiments). **f**–**i**, Relative mRNA expression for pro-inflammatory SASP components (*IL1A*, *IL1B*, *IL8*, *CCL20*) assessed by RT**–**qPCR­ after 4 d of 4OHT treatment with the indicated siRNAs (siControl, siS6K1_2, siS6K1_4, siS6K2_4 and siS6K2_5 n = 4 for *IL1A*, *IL1B* and *IL8* and *n* = 3 for *CCL20*; siS6K1/2 and siC/EBPβ *n* = 3 for *IL1A*, *IL1B*, *IL8* and *CCL20*) in IMR90 ER:RAS cells. mRNA expression was normalized to the *Rps14* housekeeping gene. *n* denotes independent experiments. Data are expressed as mean ± s.e.m. Statistical significance was calculated using one-way ANOVA with Dunnett’s multiple comparison test (**c**–**i**). **j**, Immunoblot images of a single experiment of S6K1, S6K2, IL-1β, IL-8 and GAPDH after 7 d of 4OHT treatment with the indicated siRNAs in IMR90 ER:RAS cells. IL-8 and GAPDH (loading control) were run on the same blot. S6K1, S6K2 and IL-1β were run on separate blots; therefore, GAPDH served as a sample preparation control for those blots. Source data

**Extended Data Fig. 6:**
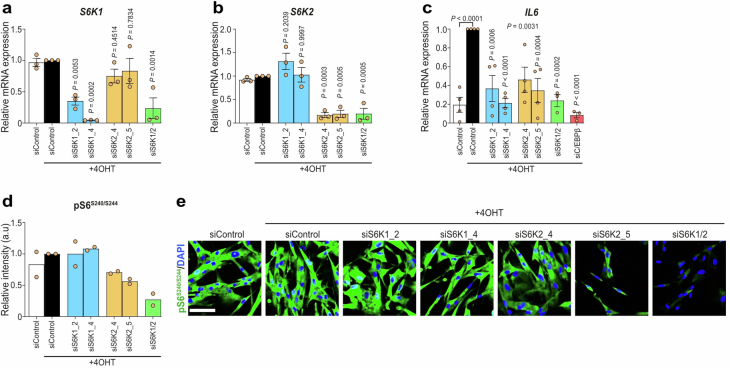
Confirmation of S6K1/2 depletion or inhibition in IMR90 ER: RAS fibroblasts. IMR90 fibroblasts were stably transduced with the pLNC-ER:RAS retroviral vector and treated with 4-hydroxytamoxifen (4OHT) for senescence induction. **a-c**. IMR-90 ER:RAS cells were reverse transfected with either Allstars (scrambled sequence - siControl) or the indicated siRNAs. Cells were treated with or without 4OHT on the following day to induce senescence. Relative mRNA expression for *S6K1* (**a**)*, S6K2* (**b**) and *Il6* (**c**) was assessed by RT-qPCR­ following 4 days of 4OHT treatment with the indicated siRNAs in IMR90 ER: RAS cells. *S6K1* and *S6K2:* n = 3 for all conditions. *IL6:* n = 4 for siControl, siS6K1_2, siS6K1_4, siS6K2_4 and siS6K2_5 and n = 3 for siS6K1/2 and siC/EBPβ. mRNA expression was normalized to the *Rps14* housekeeping gene. **d-e**. Quantification **(d)** and representative immunofluorescence images **(e)** for phosphorylated ribosomal protein S6^S240/S244^ staining of the indicated cells (n = 2). Data are expressed as mean ± SEM. Statistical significance was calculated using one-way analysis of variance with Tukey’s multiple comparison test. Scale bar, 100 μm. Source data

To understand if the above results can be extended to human cells, we took advantage of IMR90 ER:RAS cells (Fig. 5a), a widely used model to study OIS in human fibroblasts^40^. Treatment with 4-hydroxytamoxifen (4OHT) activates RAS in these cells, inducing senescence and the SASP (Fig. 5b). To analyze the role of S6K1 and S6K2, we took advantage of two independent small interferring RNAs (siRNAs) targeting each gene (Extended Data Fig. 6a,b).

Knocking down *S6K1*, *S6K2* or both kinases did not prevent senescence growth arrest, as evaluated by measuring 5-bromo-2′-deoxyuridine (BrdU) incorporation (Fig. 5c) or by quantification of the expression of key mediators of senescence growth arrest, such as the cyclin-dependent kinase inhibitors (CDKIs) p16^INK4a^ and p21^CIP1^ (Fig. 5d,e). Interestingly, transcriptional induction of multiple SASP components (*IL1A*, *IL1B*, *IL8*, *CCL20* and IL6; Fig. 5f–i and Extended Data Fig. 6c) was reduced upon knockdown of *S6K1* and/or *S6K2*, with the greatest effect observed with combined depletion. Immunoblot analysis further confirmed that knocking down *S6K1* and *S6K2* resulted in reduced SASP expression (Fig. 5j).

Ribosomal protein S6 is the best-known target for S6K1 and S6K2. S6Ks phosphorylate S6 on serine residues 240 and 244 (ref. ^7^). Knocking down of both *S6K1* and *S6K2* resulted in a decrease of S6^S240/S244^ phosphorylation as assessed by immunofluorescence (IF) (Extended Data Fig. 6d,e). Interestingly, individual knockdown of S6K1 and S6K2 had no or minimal effect on S6^S240/S244^ phosphorylation (Extended Data Fig. 6d,e), suggesting that S6^S240/S244^ phosphorylation might not explain the inhibitory effects on the SASP observed with S6K1 and/or S6K2 knockdown.

**Extended Data Fig. 7:**
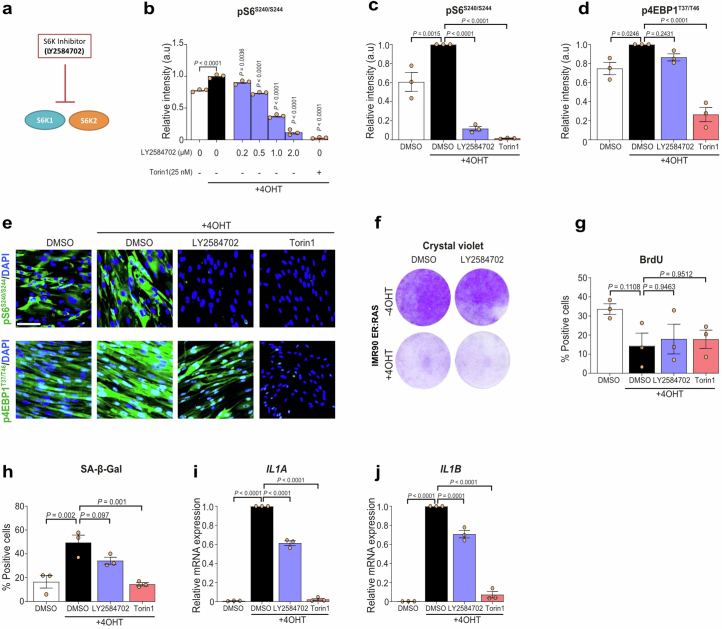
Inhibition of S6K1 and S6K2 in IMR90 ER:RAS fibroblasts undergoing RAS-induced senescence. **a**. Schematic depicting LY2584702 inhibiting both S6K1 and S6K2. **b**. IMR90 ER:RAS cells were treated with or without 4OHT in the presence of DMSO, LY2584702 (0.2-2 μM) or Torin1 (25 nM). Quantification of immunofluorescence staining for phosphorylated ribosomal protein S6^S240/S244^. n = 3 biological replicates of a single experiment. **c-e**. Quantification (**c-d**) and representative immunofluorescence images (**e**) for phosphorylated ribosomal protein S6^S240/S244^ and phosphorylated eukaryotic translation initiation factor 4E-binding protein 1 (4EBP1) following 7 days of treatment with or without 4OHT in the presence of DMSO, LY2584702 (2 μM) or Torin1 (25 nM). n = 3 independent experiments. **f**. IMR90 ER:RAS cells were treated with or without 4OHT in the presence of DMSO or LY2584702 (2 μM). Cell proliferation was assessed by colony formation assay (crystal violet staining) following 13 days in culture. **g-h**. IMR90 ER:RAS cells were treated with or without 4OHT in the presence of DMSO, LY2584702 or Torin1. Quantification of IF staining for BrdU incorporation **(g)** or senescence-associated beta-galactosidase (SA-β-Gal, **h**) following 7 days in culture. n = 3 independent experiments. **i-j**. IMR90 ER: RAS cells were treated with or without 4OHT in the presence of DMSO, LY2584702 or Torin1. Relative mRNA expression for *IL1A* **(i)** and *IL1B* **(j)** was assessed by RT-qPCR following 6 days of 4OHT treatment. mRNA expression was normalized to the *Rps14* housekeeping gene. n = 3 independent experiments. Data are expressed as mean ± SEM. Statistical significance was calculated using one-way analysis of variance with Dunnett’s multiple comparison test. Scale bar, 100 μm. Source data

To further investigate this point, we took advantage of the S6K inhibitor LY2584702 (Extended Data Fig. 7a). Treatment with LY2584702 resulted in a dose-dependent inhibition of pS6^S240/S244^ but not 4EBP1 phosphorylation (Extended Data Fig. 7b–e). Treating IMR90 ER:RAS cells with LY2584702 did not rescue the senescence growth arrest as observed in colony formation assays or measuring BrdU incorporation, although it resulted in a trend of fewer cells positive for SA-β-gal, similar to what was previously observed using the mTOR inhibitor Torin1 (ref. ^25^) (Extended Data Fig. 7f–h). Treatment with the S6K inhibitor caused a slight reduction of expression of SASP components, such as IL1A or IL1B, not similar to that observed with mTOR inhibition or depletion of *S6K1* and/or *S6K2* (Extended Data Fig. 7i,j). The above results suggest that the knockdown of S6Ks in human cells decreased the production of pro-inflammatory SASP components without preventing senescence.

### Transcriptional analysis shows that S6K1 regulates inflammatory pathways

**Fig. 6:**
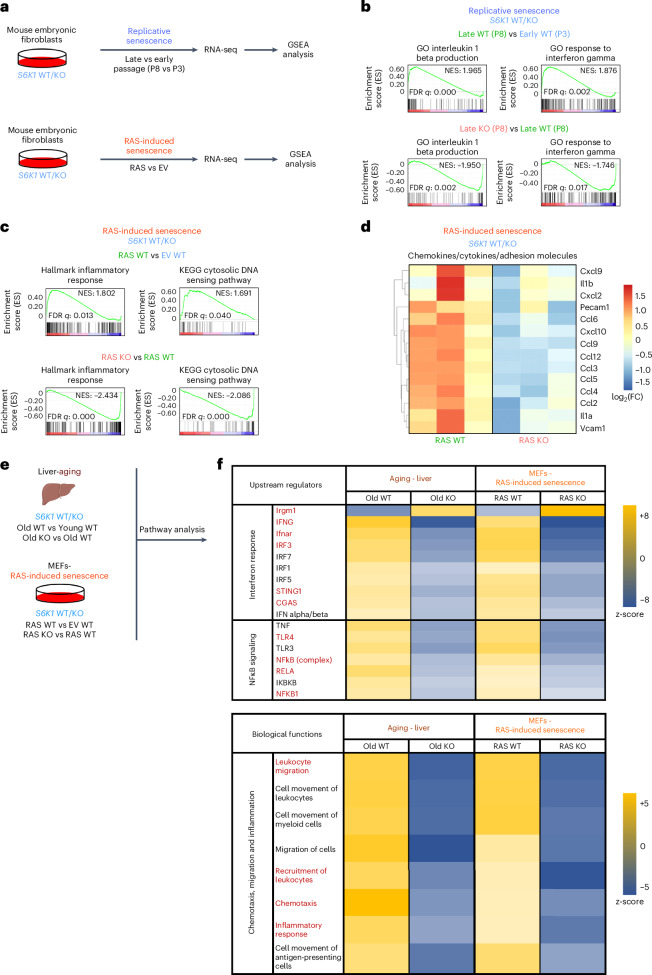
Transcriptional analysis shows that S6K1 regulates inflammatory pathways. **a**, Experimental scheme. MEFs from *S6K1* WT/KO embryos were assessed for replicative senescence or RAS-induced senescence. Samples underwent subsequent RNA-seq and GSEA. **b**. GSEA of early *S6K1* WT (passage 3), late *S6K1* WT (passage 8) and late *S6K1* KO (passage 8) MEFs. **c**, GSEA of *S6K1* WT MEFs expressing an EV, *S6K1* WT MEFs expressing RAS^G12V^ or *S6K1* KO MEFs expressing RAS^G12V^. **d**, Heatmap illustrating the gene expression pattern of key pro-inflammatory SASP factors involved in RAS-induced senescence. Left, comparison of *S6K1* WT MEFs expressing RAS^G12V^ (*n* = 3) with *S6K1* WT MEFs expressing EV (*n* = 3). Right, comparison of *S6K1* KO MEFs expressing RAS^G12V^ (*n* = 3) with *S6K1* WT MEFs expressing RAS^G12V^ (*n* = 3). **e**, Schematic of combined pathway analysis of the aging cohort and in MEFs undergoing RAS-induced senescence of the indicated comparisons to identify common upstream regulators and biological functions. **f**, Top, assessment of common upstream regulators of the SASP in *S6K1* KO mice in the aging liver and *S6K1* KO MEFs undergoing RAS-induced senescence. Bottom, assessment of biological functions that are commonly regulated in *S6K1* KO mice in the aging liver and in *S6K1* KO MEFs undergoing RAS-induced senescence. FC, fold change; FDR, false discovery rate; NES, normalized enrichment score; P, passage.

**Extended Data Fig. 8:**
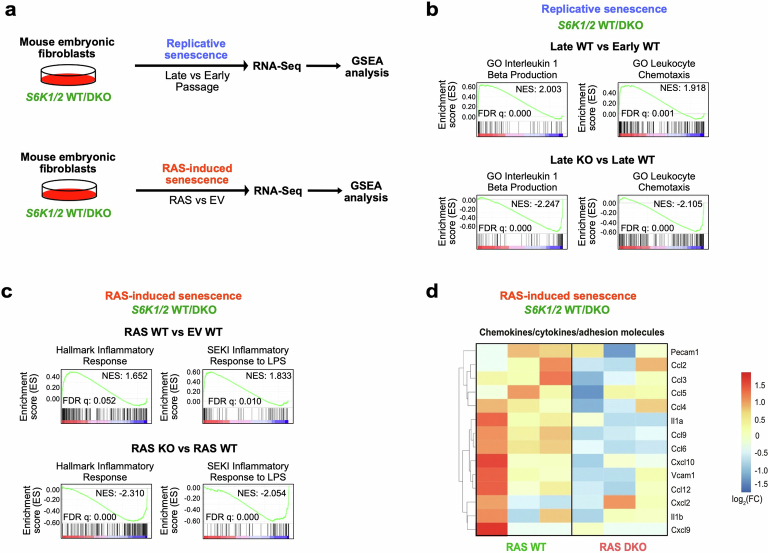
Transcriptional analysis shows that S6K1/2 regulates inflammatory pathways. **a**. Experimental scheme. Mouse embryonic fibroblasts (MEFs) from S6K1/2 WT/DKO embryos were assessed for replicative senescence or RAS-induced senescence. Samples underwent subsequent RNA-sequencing and gene-set enrichment analysis (GSEA). **b**. GSEA of early S6K1/2 WT (passage 3), late S6K1/2 WT (passage 8) and late S6K1/2 DKO (passage 8) MEFs. **c**. GSEA of S6K1/2 WT MEFs expressing an empty vector (EV), S6K1/2 WT MEFs expressing RAS^G12V^ or S6K1/2 DKO MEFs expressing RAS^G12V^. **d**. Heatmap illustrating the gene expression pattern of key proinflammatory SASP factors involved in RAS-induced senescence. **Left:** comparison of S6K1/2 WT MEFs expressing RAS^G12V^ (n = 3) with S6K1/2 WT MEFs expressing EV (n = 3). **Right:** comparison of S6K1/2 DKO MEFs expressing RAS^G12V^ (n = 3) with S6K1/2 WT MEFs expressing RAS^G12V^ (n = 3). NES: normalized enrichment score. FDR: false discovery rate. WT: wild-type. DKO: double knockout.

To further analyze the relationship between S6K1 and the inflammatory SASP, we carried out RNA-seq analysis of MEFs undergoing serial passage or RAS-induced senescence (Fig. 6a). GSEAs showed that signatures related to inflammatory cytokines and/or interferon were upregulated in late-passage WT MEFs (Fig. 6b) and upon RAS expression in these cells (Fig. 6c). Interestingly, these signatures were downregulated when comparing *S6K1* KO and WT MEFs (Fig. 6b,c). A similar downregulation of inflammatory and interferon-related signatures was also observed when comparing the transcriptional profile of late-passage and RAS-expressing *S6K1/S6K2* DKO and WT MEFs (Extended Data Fig. 8a–c). In agreement with these observations, multiple pro-inflammatory SASP components were downregulated in *S6K1* KO (Fig. 6d) and *S6K1/S6K2* DKO (Extended Data Fig. 8d) MEFs undergoing RAS-induced senescence when compared to their WT counterparts. To further explore how *S6K1* loss could result in decreased expression of inflammatory mediators in vivo, we conducted Ingenuity Pathway Analysis (IPA) of the transcriptional profile of livers of old WT and *S6K1* KO mice and compared them to those of MEFs of the same genotypes undergoing RAS-induced senescence (Fig. 6e). Biological function analysis confirmed that *S6K1* deletion in mice was associated with a downregulated inflammatory response and other associated functions, such as reduced chemotaxis or leukocyte migration (Fig. 6f). Search for upstream regulators identified cGAS and STING together with several components of the IFN (for example, IFNγ, Ifnar and IRF3) and NF-κB (for example, TLR4, NFkB, RELA and NFKB) signaling pathways (Fig. 6f). In summary, the above results suggest that S6K1-mediated modulation of different signaling pathways, including cGAS/STING, IFN and NF-κB, might explain the decreased expression of pro-inflammatory factors in cells and mice of *S6K1* KO genotype observed during aging and senescence.

### S6K1 regulates senescence surveillance

**Fig. 7:**
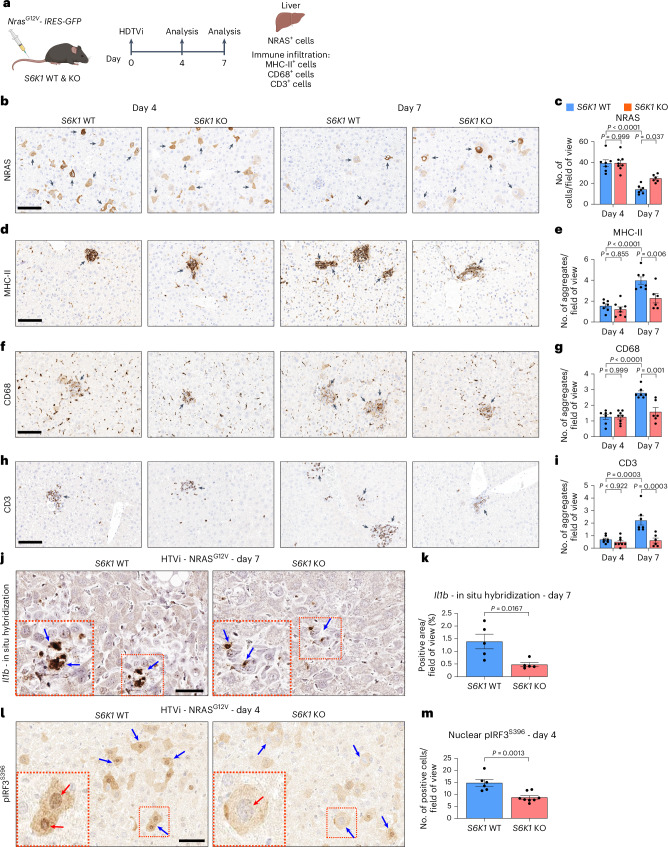
S6K1 regulates senescence surveillance. **a**, Experimental scheme. HDTVi-based co-delivery of an *Nras*^*G12V*^ transposon construct and a transposase expression vector into mouse livers (day 0). Mice were euthanized 4 d or 7 d after HDTVi to assess senescence surveillance. **b**–**i**, Immunohistochemistry staining for NRAS (**b**), MHC-II (**d**), CD68 (**f**) and CD3 (**h**) and the corresponding quantification (**c**,**e**,**g**,**i**) of livers from day 4 *S6K1* WT (*n* = 7) and KO (*n* = 8) mice and in day 7 *S6K1* WT (*n* = 7) and KO (*n* = 6) mice. Scale bar, 100 μm. **j**,**k**, In situ hybridization for *Il1b* mRNA (**j**) and quantification (**k**) of livers from day 7 *S6K1* WT (*n* = 5) and KO (*n* = 5) mice. Scale bar, 50 μm. **l**,**m**, Immunohistochemistry staining for pIRF3^S396^ (**l**) and quantification (**m**) of livers in day 4 *S6K1* WT (*n* = 6) and KO (*n* = 8) mice. Scale bar, 50 μm. Data are expressed as mean ± s.e.m. Statistical significance was calculated using two-way ANOVA with Tukey’s multiple comparison test (**c**,**e**,**g**,**i**) or by two-tailed Student’s *t*-test (**k**,**m**). *n* denotes individual mice. Source data

To confirm that S6K1 regulates inflammatory responses, affecting leukocyte chemotaxis/migration, we took advantage of a well-described mouse model of OIS and senescence surveillance (Fig. 7a). In this model, hydrodynamic tail vein injection (HDTVi) of a transposon vector expressing oncogenic Nras (Nras^G12V^) induces OIS and triggers a SASP-dependent immune surveillance response that causes the clearance of pre-neoplastic hepatocytes^41^.

We analyzed the Nras^+^ cells present 4 d and 7 d after HDTVi in the livers of *S6K1* WT and *S6K1* KO mice. At day 4, before immune-mediated clearance of senescent cells occurs, the numbers of Nras^+^ cells were similar in mice of both genotypes. The number of Nras^+^ cells decreased in mice of both genotypes by day 7, but there was a significantly higher number of Nras^+^ cells in *S6K1* KO mice when compared to their WT counterparts at day 7 (Fig. 7b,c). These differences were likely due to impaired immune-mediated elimination of senescent cells in *S6K1* KO mice, as myeloid (MHC-II^+^ and CD68^+^) and lymphoid (CD3^+^) infiltrates observed in the liver of WT mice at day 7 were significantly lower in *S6K1* KO mice (Fig. 7d–i).

To investigate whether these differences in infiltration were due to decreased expression of immunomodulatory SASP components in the senescent hepatocytes, we performed in situ mRNA hybridization to detect transcripts for *Il1b* at day 7 (Fig. 7j). *Il1b* is one of the cytokines more robustly regulated by S6K1 and plays a critical role in activating immune surveillance of senescent cells^42^. Consistent with our previous observations, there were fewer *Il1b*^*+*^ cells upon oncogenic Ras expression in *S6K1* KO mice (Fig. 7k).

**Extended Data Fig. 9:**
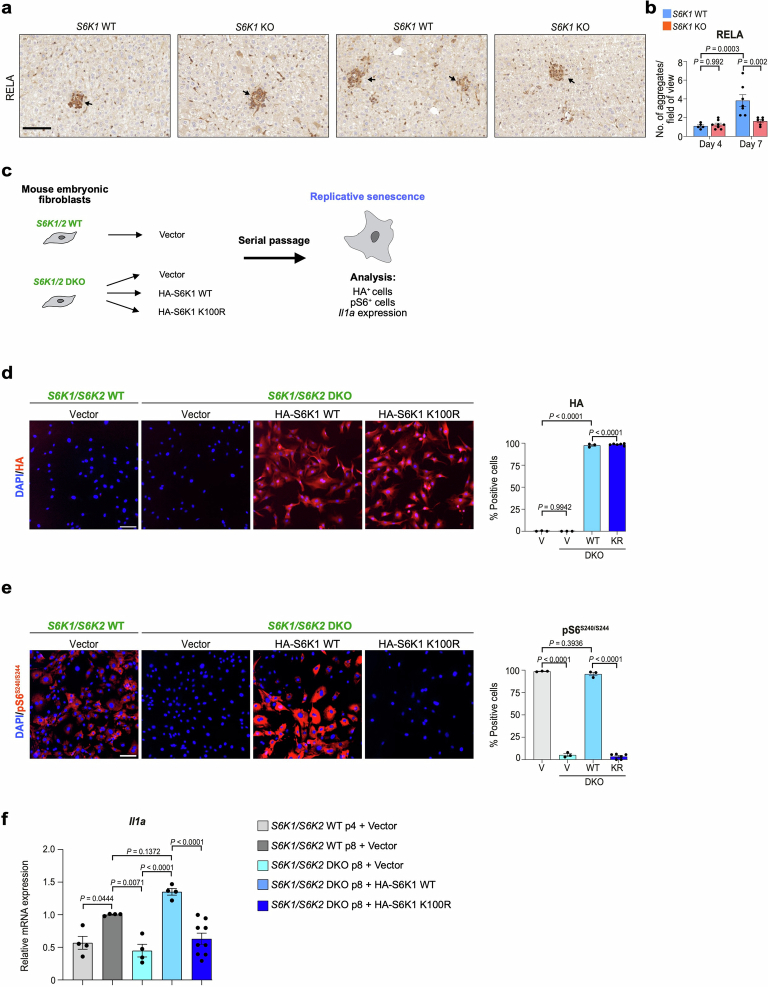
S6K1 rescues *Il1a* expression in double knockout MEFs. **a-b**. Immunohistochemistry staining for RELA (**a**) and the corresponding quantification (**b**) of livers from Day 4 S6K1 WT (n = 5), KO (n = 8) mice and in Day 7 S6K1 WT (n = 7), KO (n = 6) mice. Scale bar, 100 μm. Data are expressed as mean ± SEM. Statistical significance was calculated using two-way analysis of variance with Tukey’s multiple comparison test. n denotes individual mice. **c**. Schematic of the rescue experiment performed in S6K1/2 WT or DKO MEFs transduced with the indicated vectors and undergoing replicative senescence. **d-e**. Representative immunofluorescence (IF) images (left) and quantification (right) of the percentage of cells positive for HA (**d**) or pS6^S240/S244^ (**e**) in S6K1/2 WT or DKO MEFs infected with the indicated vectors. Scale bar: 100 µm. Data represents mean ± SEM (n = 3 for all groups, except for KR n = 6). V, vector; wt, HA-S6K1 WT; KR, HA-S6K1 K100R. Ordinary one-way ANOVA. (Sidak’s multiple comparisons test). **f**. mRNA expression levels of *Il1a* in S6K1/2 WT or DKO MEFs with the indicated vectors undergoing replicative senescence measured by qRT-PCR. Data represents mean ± SEM (n = 4 for all groups, except for KR n = 9). Statistical significance was calculated using one-way ANOVA (Sidak’s multiple comparisons test). n represents biological replicates. Source data

To understand if the reduced expression of immunomodulatory SASP components could be due to S6K1-mediated modulation of NF-κB and/or IFN signaling, we examined the expression of the NF-κB component RELA (Extended Data Fig. 9a,b) or nuclear IRF3 phosphorylated on Ser396 (pIRF3^S396^; Fig. 7l–m). Interestingly, although we could observe an increase in RELA^+^ cells at day 7 in WT mice and that decreased in *S6K1* KO mice, the signal came mostly from immune infiltrates (Extended Data Fig. 9a,b). On the other hand, we could observe many hepatocytes with nuclear pIRF3^S396^ in WT mice but mostly cytoplasmic staining in *S6K1* KO mice (Fig. 7l). The significance of this observation was confirmed upon quantification of nuclear pIRF3^S396^ (Fig. 7m). Interestingly, S6K1 (and S6K2) was previously proposed to interact with STING to activate IRF3, a key mediator of inflammatory responses, in a kinase-independent manner^27^. To understand whether the kinase activity of S6K1 is needed to explain this phenotype, we devised a rescue experiment in which WT or a kinase-dead (K100R) mutant version of HA-tagged S6K1 was expressed in S6K1/2 DKO MEFs (Extended Data Fig. 9c). IF against the HA tag confirmed the expression of both S6K1 forms (Extended Data Fig. 9d), whereas only expression of S6K1 WT restored S6^S240/S244^ phosphorylation (Extended Data Fig. 9e). Interestingly, the induction of *Il1a* observed during senescence in WT MEFs was restored in S6K1/S6K2 DKO MEFs upon expression of WT but not kinase-dead S6K1 (Extended Data Fig. 9f). The above results suggest that impaired activation of the STING/IRF3 axis in hepatocytes of *S6K1* KO mice might drive reduced immune infiltration and explain, at least in part, the decreased inflammation observed in these mice.

### Hepatocyte-intrinsic S6K signaling mediates the liver inflammatory phenotype

**Fig. 8:**
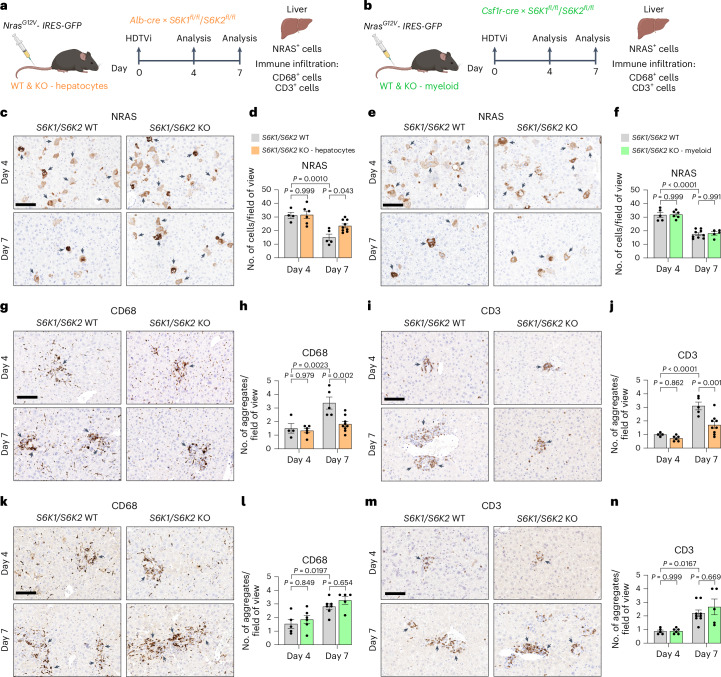
Hepatocyte-intrinsic S6K signaling mediates the liver inflammatory phenotype. **a**,**b**, Experimental scheme. HDTVi-based co-delivery of an *Nras*^*G12V*^ transposon construct and a transposase expression vector into mouse livers (day 0). Mice were euthanized 4 d or 7 d after HDTVi to assess senescence surveillance. Hepatocyte-specific *S6K1/S6K2* (**a**) or myeloid-specific *S6K1/S6K2* (**b**) KO mice or the floxed controls were used. **c**,**d**, Immunohistochemistry staining for NRAS (**c**) and the corresponding quantification (**d**) of livers from hepatocyte-specific *S6K1/S6K2* KO mice or the floxed controls. D4 WT (*n* = 4), D4 KO (*n* = 6), D7 WT (*n* = 5) and D7 KO (*n* = 9). **e**,**f**, Immunohistochemistry staining for NRAS (**e**) and the corresponding quantification (**f**) of livers from myeloid-specific *S6K1/S6K2* KO mice or the floxed controls. D4 WT (*n* = 5), D4 KO (*n* = 6), D7 WT (*n* = 9) and D7 KO (*n* = 5). **g**–**j**, Immunohistochemistry staining for CD68 (**g**) or CD3 (**i**) and the corresponding quantification (**h** and **j**) of livers from hepatocyte-specific *S6K1/S6K2* KO mice or the floxed controls. D4 WT (*n* = 4), D4 KO (*n* = 6), D7 WT (*n* = 5) and D7 KO (*n* = 9). **k**–**n**, Immunohistochemistry staining for CD68 (**k**) or CD3 (**m**) and the corresponding quantification (**l** and **n**) of livers from myeloid-specific *S6K1/S6K2* KO mice or the floxed controls. **l**, D4 WT (*n* = 5), D4 KO (*n* = 6), D7 WT (*n* = 7) and D7 KO (*n* = 5). **n**, D4 WT (*n* = 5), D4 KO (*n* = 6), D7 WT (*n* = 9) and D7 KO (*n* = 5). Data are expressed as mean ± s.e.m. Statistical significance was calculated using two-way ANOVA with Tukey’s multiple comparison test. *n* denotes individual mice. Scale bar, 100 μm. D, day. Source data

**Extended Data Fig. 10:**
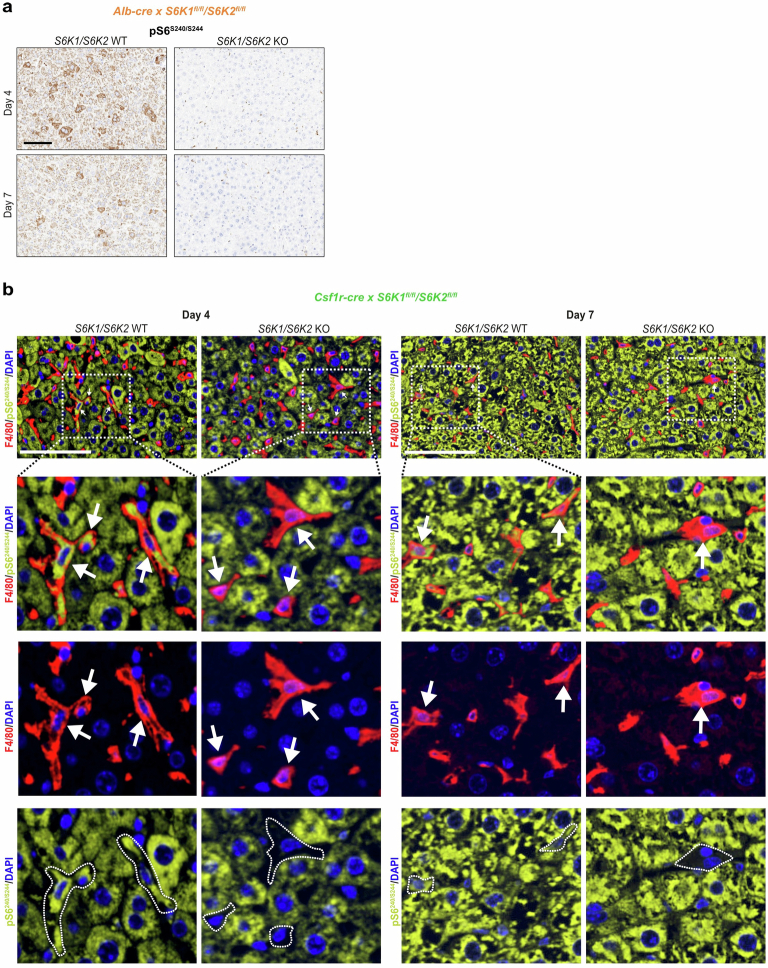
Confirmation of deletion in hepatocyte-specific or myeloid-specific *S6K1/S6K2* knockout mice. Hydrodynamic tail vein injection (HDTVi)-based co-delivery of an *Nras*^*G12V*^ transposon construct and a transposase expressing vector into mouse livers (day 0). Mice were sacrificed 4 or 7 days following HDTVi to assess senescence surveillance. Hepatocyte-specific *S6K1/S6K2* **(a)** or myeloid-specific *S6K1/S6K2* **(b)** KO mice or the floxed controls were used. **a**. Immunohistochemistry images for pS6^S240/S244^ staining in hepatocyte-specific *S6K1/S6K2* WT or KO mice. Scale bar: 100 µm. **b**. Immunofluorescence staining for F4/80 or pS6^S240/S244^ staining in myeloid-specific *S6K1/S6K2* WT or KO mice. Scale bar: 100 µm. Both **(a)** and **(b)** were single experiments with the n numbers indicated in Fig. 8.

Next, we explored whether loss of S6K signaling in hepatocytes as opposed to immune cells underlies the reduced inflammatory phenotype in the liver in vivo. To this end, we expressed oncogenic Nras^G12V^ using HDTVI in hepatocyte-specific S6K1/2 KO mice using Alb-Cre or mice lacking S6K1/2 in macrophages achieved using Csf1r-cre (Fig. 8a,b). We observed an almost complete abrogation of S6^S240/S244^ phosphorylation in the livers of hepatocyte-specific S6K1/2 KO mice (Extended Data Fig. 10a). Likewise, loss of S6^S240/S244^ phosphorylation could be seen in the liver-resident macrophage population (Kupffer cells) in livers of S6K1/2×Csf1r-cre mice (Extended Data Fig. 10b). In liver sections of the hepatocyte-specific *S6K1/2* KO mice at day 4, the numbers of Nras^+^ cells were similar to those seen in WT mice. By day 7, there was a significantly higher number of Nras^+^ cells in the hepatocyte-specific *S6K1/2* KO mice when compared to their WT counterparts (Fig. 8c,d). Therefore, hepatocyte-specific S6K1/2 KO mice recapitulated the phenotype seen in global S6K1 KO mice. In contrast, in the myeloid-specific S6K1/2 KO mice, the numbers of Nras^+^ cells were similar at both day 4 and day 7, indicating an equivalent clearance of senescent cells (Fig. 8e,f). Consistent with the data from the global S6K1 mice, in the hepatocyte-specific *S6K1/2* KO mice, myeloid (CD68) and lymphoid (CD3) cell infiltrates were reduced at day 7 compared to control mice (Fig. 8g–j). In contrast, in the myeloid S6K1/2 KO mice, no reduction in immune cell infiltration was seen at day 7 (Fig. 8k–n). Taken together, these studies indicate that hepatocyte-intrinsic S6K signaling mediates the immune activation phenotype that is ameliorated by S6K deletion and that S6K signaling in myeloid cells per se does not directly affect this phenotype.

In conclusion, we observed that S6K1 regulates age-related inflammation (inflammaging) and senescence surveillance through the modulation of key pro-inflammatory chemokines/cytokines. Given that chronic inflammation is a driver of many age-related pathologies, these results may contribute to explaining why *S6K1* KO mice are long lived and show improved healthspan.

## Discussion

Inhibition of mTOR signaling, including the key effector S6K1, extends lifespan in an evolutionarily conserved manner and increases healthspan in mice^9^. Underlying mechanisms include beneficial long-term effects on glucose homeostasis, adipose tissue biology and effects upon key hormone and energy-sensing signaling pathways^9,12,13,17^. In the current study, we demonstrate that loss of S6K signaling in the liver has marked anti-inflammatory effects and attenuates various age-related hepatic liver pathologies. This blockade of S6K signaling appears to act, at least in part, via changes in age-related inflammation (inflammaging) rather than altering senescence per se, with concomitant beneficial effects on associated phenotypes, such as age-related fibrosis. Furthermore, our studies aimed at further unraveling the cellular and molecular mechanisms at work demonstrate that deletion of S6Ks intrinsically impairs the production of pro-inflammatory cytokines in both mouse and human cells and has a broad effect on the senescence-associated inflammatory profile and immune cell recruitment. Interestingly, our data echo a recent study conducted mostly on *Drosophila* linking S6K with age-related inflammation through endolysosomal regulation^43^. At a molecular level, our data suggest that, at least in part, the effects observed in the liver are related to S6K1 activation of STING/IRF3, a key modulatory axis of immune activation. Although previous work suggested that S6K can activate STING/IRF3 in a kinase-independent fashion^27^, our work suggests that S6K1 regulation of inflammatory factors might depend on its kinase activity.

Increased accumulation of senescent cells is a recognized feature of aging in the mouse liver, although its precise origin and its pathophysiological impact remain to be determined^31,35^. Work by us and others has shown that mTOR inhibitors prevent the induction of the pro-inflammatory SASP while not affecting senescence growth arrest, an effect in part mediated by 4EBP^25,26^. However, these studies did not specifically investigate the role of S6Ks, even though the loss of S6K1 has beneficial effects on aging and related liver phenotypes^9,32^. Our current studies demonstrate in vivo, and using multiple cellular models, that depletion of S6K1 and S6K2 (singly or in combination) does not alter the accumulation of senescent cells or the senescence response per se but, rather, selectively affects their pro-inflammatory properties.

We show that loss of S6K signaling has marked effects on the SASP, with a profound reduction in a subset of inflammatory markers. In the liver of old S6K1 KO mice, several inflammatory cytokines showed reduced expression, which was associated with lowered immune cell infiltration. Aging in the liver is associated with low-grade inflammation, which may, in part, be related to calorie and macronutrient intake^31,35^. Accumulation of senescent cells in the liver was reported to promote hepatic fat accumulation and steatosis features of liver aging, and ablation of these cells ameliorates this phenotype^35^. Our findings suggest that the primary driver of the beneficial effects of the removal of senescent cells upon late-life metabolic dysfunction in the liver stems from the abrogation of the pro-inflammatory profile engendered by these cells.

Systemic deletion/inhibition of S6K1 and/or S6K2 has beneficial effects upon lipid accumulation in the liver in states of over-nutrition as well as having beneficial effects upon a range of age-related liver phenotypes^9,44,45^. However, due to the crosstalk between the liver and key metabolic tissues, such as adipose tissue and skeletal muscle, and the known effects of S6K1 deletion in these tissues, it is unclear whether the beneficial effects of this manipulation stem from liver-intrinsic effects. Studies in mice with virally mediated loss of liver S6K demonstrate beneficial systemic metabolic effects^32^, although the role of senescence and the impact upon longevity were not studied. In our current studies, we show that, during OIS, the loss of hepatocyte-specific S6K signaling recapitulates the phenotype seen in mice with global deletion of S6K1, namely reduced immune infiltration but with similar levels of senescent cells. We also demonstrate in vitro that BMDMs derived from global S61 KO mice are not intrinsically impaired. More importantly, myeloid-specific *S6K1* deletion does not attenuate immune cell infiltration and senescence surveillance in the liver during OIS. Together, these data indicate that S6K signaling in senescent hepatocytes rather than in immune cells mediates the immune activation phenotype that is ameliorated by S6K deletion.

Our previous studies demonstrated that global deletion of S6K1 results in beneficial effects on longevity and healthspan^9^. S6K1 belongs to a family of S6Ks, which also includes S6K2, which remains an understudied signaling component^46^. S6K1 and S6K2 share common substrates but also have specific ones^7^. Combined global loss of S6K1 and S6K2 protects mice from the negative effects of a high-fat diet^44^, but the relative contribution of each kinase to processes such as senescence and the SASP was unknown.

Our in vitro studies suggest that, although genetic knockdown of S6K1 resulted in alterations in a range of parameters, such as the reduction in inflammatory markers, deletion of S6K2 alone had heterogeneous but more limited effects. An exception was in OIS in human cells where knockdown of S6K2 had significant effects on inflammation, suggesting that S6K2 could have specific roles in this process or play a more important role in human cells compared to mouse cells. In general, combined deletion of both kinases had the most marked effects on inflammatory mediators in both mouse and human cells and across the range of cell models of senescence that we studied. Surprisingly, LY2584702, a pharmacological inhibitor of S6K^47^, which was effective in blocking S6K action as judged by the phosphorylation of S6, had modest effects on OIS-induced inflammation. Although LY2584702 has been shown capable of mitigating diet-induced hepatosteatosis^45^, our results suggest that a PROTAC derivative able to degrade S6K could be a more effective alternative.

IRF3 is also involved in DNA damage associated with cellular senescence^48^ and induction of the SASP and might play a role in premature aging^49^. Based on these observations, we explored a potential role for IRF3 and found impaired activation of this molecule in OIS in the livers of S6K1 KO mice. This finding might underlie, in part, the reduced inflammation observed in these mice. S6Ks have multiple substrates, however, and future work will need to explore the role of these in the regulation of the SASP.

The work presented here has several limitations but also should direct future studies in this area. For example, we did not show that liver-specific deletion of S6K signaling regulates lifespan, which would require formal aging studies. However, undertaking such studies is arduous, and there are some caveats as to whether longevity studies of cell-specific deletion recapitulate the lifespan effects of global deletion of signaling molecules. Although we previously demonstrated that global deletion of insulin receptor substrate 1 extends lifespan and healthspan^50^, recently we showed that a series of cell-specific mice did not have extended lifespan, but some had age-related health benefits^51^. Despite these caveats, future studies could, therefore, be directed at understanding the effects of abrogating S6K signaling in the liver on late-age phenotypes and healthspan and also in liver regeneration or carcinogenesis. It would also be of value to determine if the loss of S6K signaling similarly impacts inflammaging beyond the liver, as this would point toward a broader effect and strengthen the idea that chronic inflammation is a key mechanism underlying the lifespan and healthspan benefits seen in global S6K1 KO mice. Future studies should also address how inhibiting S6K1 signaling contributes to the effects that different interventions targeting mTORC1 (such as rapamycin treatment, protein restriction or branched-chain amino acid restriction) have on senescence and lifespan.

In summary, our findings show that loss of S6K signaling in aging models both in vivo and in vitro attenuates senescence-associated inflammatory processes and may play an important role in the beneficial effects of attenuation of mTOR signaling on detrimental phenotypes, particularly in the aging liver.

## Methods

### Ethics

This study complied with all relevant ethical regulations and was approved and overseen by the following ethics review boards.

Liver and myeloid-specific S6K KO mouse experiments were performed according to German law and with the approval of the Regierungspräsidium Karlsruhe (G139/19). All other mouse procedures were performed under license, according to the UK Animals (Scientific Procedures) Act of 1986 and amended regulations (2012) and approved by the Imperial Collegeʼs animal welfare and ethical review body under either 70/8700 or 70/09080.

### Mice experiments

All mice were kept under specific pathogen-­free barrier conditions within individually ventilated cages on a 12-­h light/dark cycle between 21 °C and 23 °C. Mice were given ad libitum access to food and water. Mice were fed a chow (RM3 expanded, Special Diets Services) diet. *S6K1* KO and/or *S6K2* KO mice on a C57BL/6 inbred strain were described previously^9,52^.

For the aging experiment, female *S6K1* WT and KO mice were generated from heterozygous breeding pairs or trios and aged for either 90 d (young) or 600 d (old) before being euthanized.

For the HDTVi experiments, male 8–16-week-old *S6K1* WT and KO mice, as well as hepatocyte-specific or myeloid-specific *S6K1/S6K2* DKO mice and the floxed controls, were used. HDTVi was carried out to deliver transposon-based vectors as previously described^41^. All vectors were prepared with the GenElute HP Endotoxin-Free Plasmid Maxiprep Kit (Sigma-Aldrich). On day 0, 25 µg of the vector expressing *Nras*^*G12V*^ and 5 µg of the SB-13 Sleeping Beauty transposase expression vector were diluted in sterile PBS to a total volume of 2 ml (~10% body weight) before HDTVi within 10 s. Livers were collected 4 d and 7 d after the HDTVi.

### MEF generation

MEFs were prepared as previously described^53^. In brief, MEFs were prepared from 13.5-d embryos of mice bred in heterozygosity for S6K1 or S6K2. Notably, both WT and KO MEFs of the indicated genotypes were prepared from the same mother to ensure that littermates were used. MEFs generated from at least three independent mothers were used for experiments. *S6K1/S6K2* DKO MEFs were prepared by breeding mice that were both heterozygous for *S6K1* (*S6K1*^*+/−*^) and knockout for *S6K2* (*S6K2*^*−/−*^) together (that is, *S6K1*^*+/−*^;*S6K2*^*−/−*^ *× S6K1*^*+/−*^;*S6K2*^*−/−*^). WT MEFs were used as controls for the *S6K1/S6K2* DKO experiment. WT MEFs were prepared from embryos of WT mothers that were obtained at earlier stages of breeding for the generation of double deletion.

Preparation of MEFs was performed by first removal of the embryo from the uterus and yolk sac, followed by removal of the head and viscera. The remaining tissue was minced and triturated in trypsin-EDTA (0.05%, Gibco) using a scalpel and gentle pipetting and incubation at 37 °C and 5% CO_2_ for 15 min with periodic (every 5 min) resuspension. A single-cell suspension was then obtained by passing cells through a 100-μm sterile nylon cell strainer (Falcon). Cells were cultured for 3–5 d until confluence was reached and were then frozen in complete DMEM (see below) with 10% dimethyl sulfoxide (DMSO; Sigma-Aldrich).

### Chemical compounds and drug treatments

4OHT (125 nM; Sigma-Aldrich) was dissolved in DMSO. LY2584702 (2 μM, Key Organics) and Torin1 (25 nM, Tocris) were also dissolved in DMSO. Cells were treated with the indicated drugs the day after seeding. 4OHT was replenished every 4 d, and LY2584702 and Torin1 were replenished every 2 d. MEFs were treated with 5 μM etoposide (R&D Systems, 1226) for therapy-induced senescence experiments. BMDMs were treated with 100 ng ml^−1^ LPS (Sigma-Aldrich) for the macrophage activation assay.

### Plasmids and vectors

pLNC-ER:RAS retroviral vector was previously described^54^. pBABEpuro EV or MSCV-neo vectors expressing constitutively active RAS (HRAS^G12V^) were previously described^55^. EcoHelper (pCL-Eco, Addgene) was used for the retroviral infection of MEFs. For the S6K rescue experiments, HA-tagged coding sequences of either WT or K100R S6K1 rat cDNA were PCR amplified from either pRK7-HA-S6K1-WT (Addgene, 8984) or pRK7-HA-S6K1-KR (Addgene, 8985) and cloned into EcoRI and SalI site of the retroviral vector pBABE-puro (Addgene, 1764) and sequence verified.

### Cell culture and retroviral transduction

MEFs were maintained in DMEM supplemented with EmbryoMax FBS (Millipore) and 1× antibiotic-antimycotic at 37 °C and 5% CO_2_. Human IMR90 fibroblasts and human embryonic kidney 293 transformed (HEK293T) cells were obtained from the American Type Culture Collection. For proliferation and maintenance of IMR90 and HEK293T lines, cells were grown in DMEM supplemented with 10% FBS (Sigma-Aldrich) and 1× antibiotic-antimycotic (Gibco) and kept incubated at 37 °C and 5% CO_2_. Cells were cultured in the indicated medium during experiments unless otherwise stated. Guava ViaCount reagent (Millipore) and a Guava cytometer (Millipore) were used to assess cell number and viability. Cells were routinely assessed for mycoplasma.

Retroviral transduction was performed as previously described^55,56^. HEK293T cells with or without GagPol expression were used for the packaging of retrovirus. For the generation of IMR90 ER:RAS^G12V^ fibroblasts, transfection was performed in 10-cm dishes using HEK293T cells with GagPol expression, pLNC-ER:RAS vector and packaging vectors using 1 mg ml^−1^ linear 25-kDa polyethyleneimine (PEI; Polysciences). Twenty-four hours after transfection, the medium was replaced with fresh 6 ml (to concentrate the virus) of complete DMEM, and transfection efficiency was monitored by expression of mCherry using an Olympus CKX41 inverted light microscope. Human IMR90 fibroblasts were seeded at a density of 10^6^ per 10-cm dish on the same day. Forty-eight hours after transfection, the viral supernatant was filtered (0.45 μm), supplemented with 4 μl of 8 mg ml^−1^ polybrene and added to IMR90 fibroblasts for 3 h. Two additional rounds of transduction were carried out before replacing with fresh complete DMEM. Forty-eight to seventy-two hours after transduction, cells were split and selected with Geneticin (400 μg ml^−1^).

For the generation of MEFs expressing either EV or constitutively expressing HRAS^G12V^, transfection was performed as above with a few differences. Transfection was carried out using HEK293T cells in 10-cm dishes with viral vector, EcoHelper (pCL-Eco, Addgene), and packaging vectors using 1 mg ml^−1^ 25-kDa PEI. In total, 1.5 × 10^6^ MEFs were seeded for transduction. Transduction was carried out by pooling the EV or HRAS^G12V^ supernatant together and adding an equal amount of virus titer (6 ml) to each 10-cm MEF dish. Only a single 8-h round of transduction was carried out. Forty-eight to seventy-two hours after transduction, MEFs were selected with 3 μg ml^−1^ puromycin, and transduction efficiency for mCherry was assessed by flow cytometry using Guava EasyCyte (Millipore). A transduction efficiency of 95% or greater was achieved.

### Generation and stimulation of BMDMs

Tibia and femurs from 10-week-old *S6K1* WT and KO mice were flushed with PBS + 2% FBS using a 25-gauge needle (0.5 × 16 mm) to collect the bone marrow. Cells were spun down at 1,200 r.p.m. for 5 min and resuspended in 20% L929 conditioned medium (CM) diluted in DMEM-F12 Ham supplemented with heat-inactivated HyClone FBS (GE Healthcare) and 1× antibiotic-antimycotic (Gibco). Cells were passed through a 0.70-μm pore cell strainer, counted and plated in 10-cm dishes. BMDMs were differentiated using 20% L929 CM and DMEM-F12 Ham as described above for 7 d. BMDMs were counted and seeded on day 7 at a density of 5 × 10^5^ in 6-cm dishes in 5 ml of 20% L929 with DMEM-F12 Ham as described above. On the following day, the medium was replaced, and cells were stimulated with ultra-pure 100 ng ml^−1^ LPS (Sigma-Aldrich) for 6 h.

### Reverse transfection of siRNAs

Lyophilized siRNAs targeting *S6K1* or *S6K2* were obtained from Qiagen in FlexiTubes with a preference for verified siRNA sequences. siRNAs were first reconstituted in RNase-free water to a concentration of 1 μM and aliquoted. See Supplementary Table 1 for siRNA sequences.

For RNA analysis, 1.2 × 10^5^ (for growing cells that will not be given 4OHT) or 2.4 × 10^5^ IMR-90 ER:RAS fibroblasts in suspension were reverse transfected with the indicated siRNAs in a 6-cm dish to a final volume of 4 ml in DMEM with 10% FBS but without antibiotic. The transfection mix consisted of 4 μl of DharmaFECT1 (GE Healthcare), 144 μl of 1 μM siRNA (35 nM final concentration) and 700 μl of plain DMEM. Each transfection mix was briefly vortexed and incubated at room temperature for 30 min before cell seeding. The transfection medium was replaced with fresh complete medium with or without 4OHT 16 h later once cells had adhered. AllStars scrambled siRNA served as a negative control.

For high-content IF analysis in a 96-well format, 1.75 × 10^3^ (for day 5 analysis) or 1.25–1.5 × 10^3^ (for day 8 analysis) IMR90 ER:RAS fibroblasts in suspension were reverse transfected with the indicated siRNAs in a final volume of 100 μl in DMEM with 10% FBS but without antibiotic. The transfection mix consisted of 0.1 μl of DharmaFECT1 (GE Healthcare), 3.6 μl of 1 μM siRNA (35 nM final concentration) and 17.5 μl of plain DMEM. Each transfection mix was briefly vortexed and incubated at room temperature for 30 min before cell seeding. The transfection medium was replaced with fresh complete medium with or without 4OHT 16 h later once cells had adhered. AllStars scrambled siRNA served as a negative control.

### MEF serial passage

Passage 1 MEFs of the indicated genotypes were seeded (2 × 10^6^ cells) in 10-cm dishes. Cell counts were performed using the Guava cytometer (Millipore) every 4 d (a passage) until WT cells reached replicative exhaustion. Experiments were performed in 21% O_2_, and the medium was replaced every 2 d. Cumulative population doublings per passage were calculated as log_2_ (number of cells at the time of subculture / number of cells plated) and plotted against the total time in culture (passage 2 until passage 8). RNA was extracted from cells at passage 3 (young) and passage 8 (old). Cells were also seeded to assess BrdU incorporation and SA-β-gal activity at passages 2 and 8.

### Crystal violet staining

Cells were seeded (1.5–2 × 10^4^) in six-well dishes. Cells were cultured until control (growing) cells reached confluence (usually 13 d). Every 2–4 d, the medium was replenished with the appropriate drug treatments. Cells were fixed with 0.5% glutaraldehyde (v/v) (Sigma-Aldrich) and stained with 0.2% crystal violet (w/v) (Sigma-Aldrich).

### SA-β-gal staining

The SA-β-gal activity was assessed as previously described^57^. For cytochemistry assays, passage 2 and passage 8 MEFs were seeded (8 × 10^4^) in six-well dishes and fixed the following day with 0.5% glutaraldehyde (v/v) (Sigm-Aldrich). Cells were washed twice in 1 mM magnesium chloride (MgCl_2_) in PBS (pH 5.5) and incubated in X-Gal staining solution (1 mg ml^−1^; Thermo Fisher Scientific; 5 mM K_3_[Fe(CN)_6_] and 5 mM K_4_[Fe(CN)_6_×3H_2_O]) for 8 h at 37 °C. Bright-field images were acquired using an Olympus CKX41 inverted light microscope and an Olympus DP20 digital camera. The percentage of SA-β-gal^+^ (blue staining) cells was estimated by counting at least 150 cells per well.

For fluorescence assays, IMR90 ER:RAS cells (1.5–2.5 × 10^3^) were seeded in 96-well plates in triplicate and treated with the indicated drugs on the following day. Eight days later, cells were incubated with fresh DMEM containing DDAO galactoside (9*H*-(1,3-dichloro-9,9-dimethylacridin-2-one-7-yl) β-d-galactopyranoside) (Molecular Probes) for 2 h. Cells were then fixed in 4% formaldehyde solution (v/v) (Sigma-Aldrich), washed and stained with 4′,6-diamidino-2-phenylindole (DAPI, 1 μg ml^−1^) for 15 min. Fluorescence images were acquired and analyzed by high-content analysis (HCA) microscopy using an InCell Analyzer 2000 (GE Healthcare) and InCell Investigator software 2.7.3. The percentage of SA-β-gal^+^ (blue staining) cells was estimated by counting at least 1,000 cells per well.

### Assessing BrdU incorporation

BrdU incorporation was assessed as previously described^57^. In brief, BrdU incorporation was carried out by high-content microscopy and analysis. IMR90 ER:RAS fibroblasts were seeded (1–3 × 10^3^) in 96-well plates in triplicate, allowed to adhere overnight and treated with or without 4OHT (±any additional treatments) on the following day. Cells were then pulsed overnight (17 h) in BrdU (50 μM) and then fixed in 4% formaldehyde solution (v/v). Cells were permeabilized with 0.2% Triton X-100 (v/v) (Sigma-Aldrich) for 15 min and blocked with 0.5% BSA (w/v) and 0.2% fish skin gelatin (v/v) in PBS for 1 h at room temperature with gentle shaking. Fixed cells were incubated with mouse anti-BrdU primary antibody and DNAse (0.5 U μl^−1^; Sigma-Aldrich) in the presence of 1 mM MgCl_2_ in blocking solution and incubated for 30 min at room temperature. Cells were washed before incubation with goat anti-mouse Alexa Fluor 594 secondary antibody in blocking solution for 30 min. Cells were washed before incubation with 1 μg ml^−1^ DAPI. Plates were then analyzed by high-content microscopy (described below).

Passage 2 and passage 8 *S6K1/S6K2* WT/DKO MEFs for the cumulative population doubling experiment were seeded (8 × 10^3^) in 96-well plates in triplicate and allowed to adhere overnight, whereas passage 2 MEFs from S6K1 WT/KO, S6K2 WT/KO and/or S6K1/S6K2 WT/DKO MEFs for the timecourse were seeded at a density of 3 × 10^3^ in 96-well plates in triplicate and allowed to adhere overnight. Cells were pulsed for 8 h with BrdU (50 μM) and processed as described above.

### IF and HCA microscopy

IF and HCA were performed as previously described^25,57^. A list of antibodies and dilutions used can be found in Supplementary Table 2. Cells were seeded in 96-well plates (Nunc, Thermo Fisher Scientific), allowed to adhere overnight, treated with the indicated drugs on the following day and fixed on the desired day of analysis. Medium was replaced every 2–4 d. Cells were fixed in 4% formaldehyde solution (v/v), permeabilized with 0.2–0.3% Triton X-100 (v/v) and blocked with 0.5% BSA (w/v) and 0.2% fish skin gelatin (v/v) in PBS. For SASP and RPS6 staining, cells were blocked in a solution containing 5% donkey serum, 0.3% Triton X-100 and 0.1% BSA. Cells were incubated with the primary antibody for 1–1.5 h at room temperature and washed, followed by incubation with the appropriate secondary antibody (Alexa Fluor 488 and/or Alexa Fluor 594, 1:750 dilution). Cells were then washed before incubation with 1 μg ml^−1^ DAPI.

Images were acquired using an automated high-throughput microscope (InCell Analyzer 2000, GE Healthcare) with a ×20 objective. Image acquisition was set up so that at least 1,000 cells per well from multiple fields were detected. The InCell Analyzer 2000 captured images in four different wavelengths (DAPI, FITC 488, Texas Red 594 and PE-Cy5–DDAOG). Experiments were performed in either duplicate or triplicate wells. InCell Investigator software 2.7.3 (GE Healthcare) was used for image processing and quantification. Nuclei segmentation and cell identification were performed using DAPI staining. Nuclei were segmented using a TopHat segmentation approach (a minimum area of 120 μm^2^). The cell area was defined either using a collar segmentation approach that placed a border of 2 μm around the DAPI staining or using cytoplasmic intensity by a multi-scale TopHat approach. The cellular expression (nuclear or cytoplasmic) of the protein of interest was calculated by quantifying the mean intensity of pixels in the desired reference channel (FITC 488 or Texas Red 594). A histogram was generated that assigned intensity values for all of the cells in a given sample. Then, a threshold filter to define the number of positive and negative cells for the given protein or signal of interest was set up by assigning a nuclear or cytoplasmic intensity value to each cell that correlates to the specific expression. Alternatively, normalized intensity values for a given staining were calculated by measuring the difference between the raw intensity and the intensity of the background (secondary antibody alone). The relative fold change to a specified condition (for example, cells treated with 4OHT) was then calculated using the normalized intensity values. The specificity of antibodies was validated with the use of robust controls (shRNAs/siRNAs, overexpression or drug inhibition).

### Immunohistochemistry and double immunofluorescence

Tissues were fixed in 4% paraformaldehyde solution (Santa Cruz Biotechnology) overnight at 4 °C, dehydrated and embedded in paraffin. Paraffin-­embedded liver sections (2 μm or 7 µm for Sirius Red staining) were processed for immunohistochemistry staining using a BOND-MAX system (Leica) as previously described^58^. After deparaffinization and rehydration, antigen retrieval was carried out with BOND citrate solution (AR9961, Leica), BOND EDTA solution (AR9640, Leica) or a BOND proteolytic enzyme kit (AR9551, Leica). Sections were then incubated with the indicated antibodies in BOND primary antibody diluent (AR9352, Leica). This was followed by incubation with secondary antibodies (Leica) and staining using a BOND Polymer Refine Detection Kit (DS9800, Leica). Whole slides were then scanned using an Aperio AT2 slide scanner (Leica) at ×20 objective. Whole slides were annotated and analyzed using Aperio ImageScope (version 12.4.0.5043, Leica) and Fiji (ImageJ version 1.52e, National Institutes of Health).

Antibodies used included anti-Ki67, rabbit, 1:200 (Thermo Fisher Scientific, RM-9106-S1); anti-CHOP, rabbit, 1:100 (Cell Signaling Technology, 5544); anti-BiP, rabbit, 1:200 (Cell Signaling Technology, 3177); anti-MHC-Il, rat, 1:500 (clone M5/114.15.2, Novus Biologicals, NBP1-43312); anti-CD68, rabbit, 1:200 (Abcam, 125212); anti-F4/80, rat, 1:250 (Linaris, T2006); anti-CD3, rabbit, 1:500 (clone SP7, Invitrogen, MA1-90582); anti-B220, rat, 1:3,000 (clone RA3-6B2, BD Biosciences, 553084); anti-CD4, rat, 1:1,000 (eBioscience, 14-9766); anti-CD42b, rabbit, 1:200 (Abcam, clone SP219, ab183345); anti-NRAS, mouse, 1:50 (Santa Cruz Biotechnology, sc-31); anti-pIRF3^S396^, rabbit, 1:300 (Bioss, BS-3195R); anti-pS6^S240/S244^, rabbit, 1:2,000 (Cell Signaling Technology, D68F8); and anti-RELA, rabbit, 1:800 (Novus Biologicals, NB100-2176).

For double IF, anti-pS6^S240/S244^, rabbit, 1:500 (Cell Signaling Technology, D68F8) and anti-F4/80, rat, 1:250 (Linaris, T2006) were used. AKOYA Biosciences Opal Fluorophore kits (Opal 540, FP1487001KT and Opal 620, FP1495001KT) were used and counter-stained with AKOYA 10X Spectral DAPI (FP1490) according to the manufacturer’s instructions. Whole slides were then scanned using a NanoZoomer S60 Hamamatsu digital slide scanner. Images were analyzed using NDP.view 2.7.25 software.

### Calculating nuclear parameter-based TSSs

Senescence in H&E-stained liver sections was calculated as described in ref. ^39^, following scripts deposited in https://github.com/Sen-Lab-LMS/Senescence_nuclear_features, which is archived in Zenodo with the identifier 10.5281/zenodo.10499895. In brief, H&E-stained slides were imaged with an Aperio AT2 slide scanner (Leica), and nuclear parameters were extracted using QuPath software (version 0.3.2). These parameters were then used to calculate cell senescence scores (CSSs). Finally, the distribution of CSS values on whole liver slide sections determined the basis for calculating the TSS.

### RNA in situ hybridization

RNA in situ hybridization in 6-μm liver sections was performed using an RNAscope 2.5 assay (FFPE and 2.5 HD Brown Assay) from Advanced Cell Diagnostics (ACD), according to the manufacturer’s protocol. Probes for Mm-*Il1b* (cat. no. 316891), the housekeeping gene Mm-*Ppib* (positive control, cat. no. 313911) and the bacterial gene *dapB* (negative control, cat. no. 310043) were purchased from ACD. Signal detection was carried out by DAB staining. Slides were counterstained with hematoxylin before mounting, and then whole digital slides were acquired using the Aperio AT2 slide scanner (Leica) at ×40 objective.

### Peripheral complete blood count

Whole blood was collected from the tail vein using citrate as an anti-coagulant and diluted using saline to a volume of at least 200 μl. Complete blood counts were then obtained using a Sysmex XE-2100 automated cell counter (Sysmex Corporation).

### Immunoblotting

Snap-frozen liver tissues (30 mg) were lysed and homogenized in RIPA lysis and extraction buffer (50 mM Tris pH 8, 150 mM NaCl, 1% Triton X-100, 0.5% Na-Doc, 0.1% SDS and 1 mM EDTA) containing cOmplete, Mini, EDTA-free protease and phosphatase inhibitors (Roche) using a T10 basic ULTRA-TURRAX homogenizer (IKA). Lysates were incubated on ice for 10 min for lysis before being spun at 16,000*g* (4 °C) for 10 min. The supernatant (protein extracts) was collected. Cells were washed twice in ice-cold PBS before lysis in RIPA buffer.

Protein concentration was measured using a DC Protein Assay (Bio-Rad) according to the manufacturer’s protocol, and samples were denatured in Laemmli sample buffer. Samples were loaded into a 4–15% Mini-PROTEAN TGX Precast Gel (Bio-Rad). Immunoblotting was carried out as previously described^59^. See Supplementary Table 2 for the list of antibodies and dilutions used.

### Gene expression analysis

Total RNA was extracted from liver tissues or cells using TRIzol reagent (Ambion) and an RNeasy Mini Kit (Qiagen) as previously described^57^. cDNA synthesis was carried out using a SuperScript II reverse transcriptase kit (Invitrogen) with dTNPs and random hexamers according to the manufacturer’s protocol. PCR reactions were carried out in a CFX96 Real-Time PCR system (Bio-Rad) using Power SYBR Green Master Mix (Applied Biosystems). Gene expression was normalized to ribosomal protein S14 (RPS14) housekeeping genes unless stated otherwise and analyzed by the comparative Ct method. See Supplementary Table 3 for the list of primers.

### RNA-seq and analysis

RNA purity and integrity were first determined with an Agilent 2100 Bioanalyzer and an Agilent RNA 6000 Nano Kit, and an RNA integrity (RIN) score higher than 7 was used for quality assurance.

The cDNA library was then prepared from 500 ng of total RNA using the TruSeq Stranded mRNA library kit (Illumina) according to the manufacturer´s protocol. The quality of the library was assayed with the Agilent 2100 Bioanalyzer with an HS DNA chip, and the DNA concentration was measured with a Qubit (Life Technologies). The cDNA library was sequenced with a HiSeq 2500 (Illumina) with single-end, 50-bp reads for a minimum of 40 million reads per sample.

The data were processed using standard procedures. In brief, demultiplexing was carried out with CASAVA software (version 1.8.4), and raw reads were mapped to the mm9 genome using TopHat aligner. The ‘HTseq counts module’ was used to obtain gene-based counts. The DESeq2 Bioconductor package was used for differential expression analysis. Sample-to-sample distances were calculated using the ‘dist’ function in DESeq2. GSEA was conducted using the Broad Institute GSEA application based on Wald statistics obtained from the DESeq2 comparisons. Genes with very few read events were excluded. Distances were visualized by the ‘pheatmap’ function available in the pheatmap R package.

The Broad Institute GSEA application was used to carry out GSEA. The genes were ranked based on Wald statistics from DESeq2 comparisons. Genes with very few read counts were excluded from the analysis. Qiagen IPA was used to identify common ‘upstream regulators’ and ‘biological functions’.

### Statistical analysis and reproducibility

Data are expressed as mean ± s.e.m. unless stated otherwise. Statistical analyses were performed using GraphPad Prism 9 software. Statistical significance for comparing two groups was calculated using an unpaired Student’s *t*-test. Comparison of three or more groups with one variable was calculated using one-way ANOVA with Tukey’s or Dunnett’s multiple comparison test. Comparisons of four groups with two variables were performed using a two-way ANOVA with Tukey’s multiple comparison test. *P* values less than 0.05 were considered statistically significant. Data distribution was assumed to be normal, but this was not formally tested.

No statistical methods were used to pre-determine sample sizes. Mice were randomly allocated to either 90 d or 600 d for the aging experiment. Mice were randomly assigned to either day 4 or day 7 for the HTVi experiment. Cell culture experiments did not require randomization because the tests were compared to controls. Plates needed to be marked to ensure that the treatments were delivered to the appropriate plates (and not the control), and randomization would not be practical or feasible. Investigators were blinded to the genotype during dissection of the aging and HTVi experiments (mouse number was used for identification). Investigators were not blinded during the cell culture experiments, as identification was required to carry out correct treatments. Mice that developed age-related dermatitis or anal prolapses were excluded from the study. In rare circumstances, the immunohistochemical staining did not work in some slides, and these were excluded. For p16 and p19 expression, one of the old WT mice showed abnormally high (>100-fold) expression and was, therefore, excluded. For whole blood analysis, three mice were excluded for platelet count as they had undergone clotting. Mice in which the HTV injection failed were excluded.

### Reporting summary

Further information on research design is available in the Nature Portfolio Reporting Summary linked to this article.

## Supplementary information

Supplementary Information Index referring to the Supplementary Information and Supplementary Tables 1–3 (included as an independent Excel file).

Reporting Summary

Supplementary Tables 1–3 Contains Supplementary Tables 1–3 as different sheets in the Excel file.

## Source data

Source Data Fig. 1 Statistical source data.

Source Data Fig. 2 Statistical source data.

Source Data Fig. 3 Statistical source data.

Source Data Fig. 4 Statistical source data.

Source Data Fig. 5 Statistical source data.

Source Data Fig. 7 Statistical source data.

Source Data Fig. 8 Statistical source data.

Source Data Extended Data Fig. 1 Statistical source data.

Source Data Extended Data Fig. 3 Statistical source data.

Source Data Extended Data Fig. 4 Statistical source data.

Source Data Extended Data Fig. 5 Statistical source data.

Source Data Extended Data Fig. 6 Statistical source data.

Source Data Extended Data Fig. 7 Statistical source data.

Source Data Extended Data Fig. 9 Statistical source data.

Source Data Fig. 1b Unprocessed western blots.

Source Data Fig. 5j Unprocessed western blots.

Source Data Extended Data Fig. 5a Unprocessed western blots.

## Data Availability

RNA-seq data have been deposited in the Gene Expression Omnibus under accession codes GSE218682, GSE218683 and GSE218684.
