# Supplementary material for: Ribosomal S6 kinase 1 regulates inflammaging via the senescence secretome
Source: Nat Aging. 2024 Aug 29;4(11):1544–61. doi: 10.1038/s43587-024-00695-z (PMC11564105; doi:10.1038/s43587-024-00695-z)
Supplement: Supplementary file 1 — Index referring to the Supplementary Information and Supplementary Tables 1–3 (included as an independent Excel file). [file 43587_2024_695_MOESM1_ESM.pdf]

nature aging

Supplementary information https://doi.org/10.1038/s43587-024-00695-z

Ribosomal S6 kinase 1 regulates inflammaging via the senescence secretome

In the format provided by the authors and unedited

Supplementary information includes:

Supplementary Table 1. List of siRNA sequences used in this study.
Supplementary Table 2. List of antibodies and dilutions used for IF and WB.
Supplementary Table 3. List of RT-qPCR primers used in this study.

The three tables are included as part of the file **SupTables1to3Gallage.xlsx**
