# Supplementary material for: Ribosomal S6 kinase 1 regulates inflammaging via the senescence secretome
Source: Nat Aging. 2024 Aug 29;4(11):1544–61. doi: 10.1038/s43587-024-00695-z (PMC11564105; doi:10.1038/s43587-024-00695-z)
Supplement: Supplementary file 2 — Reporting Summary [file 43587_2024_695_MOESM2_ESM.pdf]

nature portfolio

Corresponding author(s): Jesus Gil, Dominic Withers, Mathias Heikenwalder

Last updated by author(s): Jul 16, 2024

Reporting Summary

Nature Portfolio wishes to improve the reproducibility of the work that we publish. This form provides structure for consistency and transparency in reporting. For further information on Nature Portfolio policies, see our Editorial Policies and the Editorial Policy Checklist.

Statistics

For all statistical analyses, confirm that the following items are present in the figure legend, table legend, main text, or Methods section.

n/a	Confirmed	
☐	☒	The exact sample size (n) for each experimental group/condition, given as a discrete number and unit of measurement
☐	☒	A statement on whether measurements were taken from distinct samples or whether the same sample was measured repeatedly
☐	☒	The statistical test(s) used AND whether they are one- or two-sided<br>*Only common tests should be described solely by name; describe more complex techniques in the Methods section.*
☒	☐	A description of all covariates tested
☐	☒	A description of any assumptions or corrections, such as tests of normality and adjustment for multiple comparisons
☐	☒	A full description of the statistical parameters including central tendency (e.g. means) or other basic estimates (e.g. regression coefficient) AND variation (e.g. standard deviation) or associated estimates of uncertainty (e.g. confidence intervals)
☐	☒	For null hypothesis testing, the test statistic (e.g. F, t, r) with confidence intervals, effect sizes, degrees of freedom and P value noted<br>*Give P values as exact values whenever suitable.*
☒	☐	For Bayesian analysis, information on the choice of priors and Markov chain Monte Carlo settings
☒	☐	For hierarchical and complex designs, identification of the appropriate level for tests and full reporting of outcomes
☒	☐	Estimates of effect sizes (e.g. Cohen's d, Pearson's r), indicating how they were calculated

Our web collection on statistics for biologists contains articles on many of the points above.

Software and code

Policy information about availability of computer code

Data collection	IN Cell Analyzer 2000 (GE Healthcare).<br>Aperio AT2 slide scanner (Leica, Germany).<br>Nano Zoomer S60 Hamamatsu digital slide scanner (Japan).
Data analysis	IN Cell Investigator version 2.7.3.<br>Aperio ImageScope v12.4.0.5043.<br>NDP.view 2.7.25.<br>Graphpad Prism 9.<br>Fiji (ImageJ ver. 1.52e).<br>QuPath software (v. 0.3.2).<br>CASAVA software (ver. 1.8.4).<br>Qiagen Ingenuity Pathway Analysis (IPA - Spring Release April 2022).<br>GSEA (Broad Institute).

For manuscripts utilizing custom algorithms or software that are central to the research but not yet described in published literature, software must be made available to editors and reviewers. We strongly encourage code deposition in a community repository (e.g. GitHub). See the Nature Portfolio guidelines for submitting code & software for further information.

Data

Policy information about availability of data

All manuscripts must include a data availability statement. This statement should provide the following information, where applicable:

- Accession codes, unique identifiers, or web links for publicly available datasets
- A description of any restrictions on data availability
- For clinical datasets or third party data, please ensure that the statement adheres to our policy

Source numerical data is included with the published manuscript. We have included the uncropped western blots. We have deposited in GEO the RNAseq data and included a data availability statement: RNAseq data have been deposited in the Gene Expression Omnibus (GEO) under accession codes GSE218682 , GSE218683 and GSE218684.

Human research participants

Policy information about studies involving human research participants and Sex and Gender in Research.

Reporting on sex and gender	N/A
Population characteristics	N/A
Recruitment	N/A
Ethics oversight	N/A

Note that full information on the approval of the study protocol must also be provided in the manuscript.

Field-specific reporting

Please select the one below that is the best fit for your research. If you are not sure, read the appropriate sections before making your selection.

☒ Life sciences ☐ Behavioural & social sciences ☐ Ecological, evolutionary & environmental sciences

For a reference copy of the document with all sections, see nature.com/documents/nr-reporting-summary-flat.pdf

Life sciences study design

All studies must disclose on these points even when the disclosure is negative.

Sample size	No statistical methods were used to pre-determine sample sizes in this study, but our sample sizes are similar to what we have reported previously which aimed to reach a statistical power of at least 80%.
Data exclusions	Mice that developed age-related dermatitis or anal prolapses were excluded from the study. In rare circumstances, the IHC staining did not work in some slides and these were excluded. For p16 and p19 expression, one of the Old WT mice showed abnormally high (>100 fold) expression and was therefore excluded. For whole blood analysis, 3 mice were excluded for platelet count as they had undergone clotting. Mice where the HTV injection failed were excluded.
Replication	The aging experiment was performed using two independent cohorts which behaved comparably. MEFs were isolated from at least three independent mothers and at least n=3 were used for replicative senescence and ras-induced senescence experiments. The HTVi experiments were performed once (single experiment) using sufficient n numbers to reach statistical significance. Cell culture experiments were performed with 3 biological replicates and in most cases in three independent experiments unless otherwise stated.
Randomization	Mice were randomly allocated to either 90 days or 600 days for the aging experiment. Mice were randomly assigned to either Day 4 or Day 7 for the HTVi experiment. Cell culture experiments did not require randomisation because the tests were compared to controls. Plates needed to be marked to ensure the treatments are delivered to the appropriate plates (and not the control) and randomisation would not be practical or feasible.
Blinding	Investigators were blinded to the genotype during dissection of the HTVi experiment (mouse number used for identification). Investigators were not blinded during the cell culture experiments as identification was required to carry out correct treatments. Plates needed to be marked to ensure the treatments are delivered to the appropriate plates (and not the control) and blinding would not be practical or feasible.

Reporting for specific materials, systems and methods

We require information from authors about some types of materials, experimental systems and methods used in many studies. Here, indicate whether each material, system or method listed is relevant to your study. If you are not sure if a list item applies to your research, read the appropriate section before selecting a response.

Materials & experimental systems

n/a	Involved in the study
☐	☒ Antibodies
☐	☒ Eukaryotic cell lines
☒	☐ Palaeontology and archaeology
☐	☒ Animals and other organisms
☒	☐ Clinical data
☒	☐ Dual use research of concern

Methods

n/a	Involved in the study
☒	☐ ChIP-seq
☒	☐ Flow cytometry
☒	☐ MRI-based neuroimaging

Antibodies

Antibodies used

The following antibodies were used for IHC:
anti-Ki67, rabbit, 1:200 (Thermo Scientific, RM-9106-S1); anti-CHOP, rabbit, 1:100 (Cell Signaling, #5544); anti-BiP, rabbit, 1:200 (Cell Signaling, #3177); anti-MHCII, rat, 1:500 (clone M5/114.15.2, Novus Biologicals, NBP1-43312); anti-CD68, rabbit, 1:200 (abcam, 125212); anti-F4/80, rat, 1:250 (Linaris, T2006); anti-CD3, rabbit, 1:500 (clone SP7, Invitrogen, MA1-90582), anti-B220, rat, 1:3000 (clone RA3-6B2 – BD Biosciences, 553084), anti-CD4, rat, 1:1000 (eBioscience, 14-9766); anti-CD42b, rabbit, 1:200 (Abcam, clone SP219, ab183345). anti-NRAS, mouse, 1:50 (Santa Cruz - sc-31), anti-pIRF3S396, rabbit, 1:300 (Bioss, BS-3195R), anti-pS6S240/S244, rabbit, 1:2000 (Cell Signalling – D68F8), anti-RELA, rabbit, 1:800 (Novus Biologicals, NB100-2176).

The following antibodies were used for IF and/or WB:
S6K1 (WB) 49D7 Cell Signaling #2708 1:1000
S6K2 (WB) Polyclonal Cell Signaling #14130 1:500
Phospho-RPS6 (S240/S244) (WB/IF) D68F8 Cell Signaling #5364 1:10,000-40,000 (WB) and 1:800 (IF)
Phospho-4EBP1 (T37/T46) (IF) 236B4 Cell Signaling #2855 1:750 (IF)
BrdU (IF) PRB-1 Invitrogen A21303 1:1500
p16INK4A (IF) JC-8 CRUK 1:750
p21CIP1 (IF) Polyclonal Santa Cruz SC-471 1:200
p53 (IF) DO1 Santa Cruz SC-126 1:100
IL-1α (IF) #4414 R&D Systems MAB200 1:100
IL-1β (IF) #8516 R&D Systems MAB201 1:100
IL-1β (WB) Polyclonal Santa Cruz SC-7884 1:200
IL-8 (WB) 6217 R&D Systems MAB208 1:100
GAPDH (WB) Polyclonal Abcam ab22555 1:2000
HRAS (WB) Polyclonal Santa Cruz SC-520 1:1000
HA tag (IF) Polyclonal Abcam ab9110 1:500

Secondary antibodies IF:
Alexa Fluor 488® and/or 594® - 1:750 dilution.
The AKOYA Biosciences Opal Fluorophore kits (Opal 540, FP1487001KT and Opal 620, FP1495001KT).

Validation

p16INK4a (JC-8)
https://www.scbt.com/p/p16-antibody-jc8
p21
https://www.scbt.com/p/p21-antibody-m-19
IL-1a
https://www.rndsystems.com/products/human-il-1alpha-il-1f1-antibody-4414_mab200
IL-1b
https://www.rndsystems.com/products/human-il-1beta-il-1f2-antibody-8516_mab201
IL-1b
https://www.scbt.com/de/p/il-1beta-antibody-h-153
IL-8
https://www.rndsystems.com/products/human-il-8-cxcl8-antibody-6217_mab208
GAPDH
https://www.abcam.com/gapdh-antibody-loading-control-ab22555.html
HRAS
https://www.scbt.com/de/p/h-ras-antibody-c-20
HA tag
https://www.abcam.com/en-de/products/primary-antibodies/ha-tag-antibody-chip-grade-ab9110
pIRF3
https://www.thermofisher.com/antibody/product/Phospho-IRF3-Ser396-Antibody-Polyclonal/BS-3195R
pS6
https://www.cellsignal.de/products/primary-antibodies/phospho-s6-ribosomal-protein-ser240-244-d68f8-xp-rabbit-mab/5364
S6K1
https://www.cellsignal.com/products/primary-antibodies/p70-s6-kinase-49d7-rabbit-mab/2708
S6K2
https://www.cellsignal.com/products/primary-antibodies/p70-s6-kinase-2-antibody/14130

p4eBP1
https://www.cellsignal.de/products/primary-antibodies/phospho-4e-bp1-thr37-46-236b4-rabbit-mab/2855
BrdU
http://tools.thermofisher.com/content/sfs/manuals/mp21300.pdf
Ki67
https://tools.thermofisher.com/content/sfs/brochures/D12536~.pdf
CHOP
https://www.cellsignal.com/products/primary-antibodies/chop-d46f1-rabbit-mab/5554?&print=true
BiP
https://www.cellsignal.de/products/primary-antibodies/bip-c50b12-rabbit-mab/3177?N=4294967254&Nrpp=200&fromPage=plp
MHCII
https://www.novusbio.com/products/mhc-class-ii-i-a-i-e-antibody-m5-114152_nbp1-43312
CD68
https://www.abcam.com/cd68-antibody-ab125212.html
CD3
https://www.thermofisher.com/antibody/product/CD3e-Antibody-clone-SP7-Monoclonal/MA1-90582
B220
https://www.bdbiosciences.com/en-de/products/reagents/flow-cytometry-reagents/research-reagents/single-color-antibodies-ruo/purified-rat-anti-mouse-cd45r-b220.553084
CD4
https://www.thermofisher.com/antibody/product/CD4-Antibody-clone-4SM95-Monoclonal/14-9766-82
CD42b
https://www.abcam.com/cd42b-antibody-sp219-ab183345.html
NRAS
https://www.scbt.com/p/n-ras-antibody-f155
RELA
https://www.novusbio.com/products/rela-nfkb-p65-antibody_nb100-2176

Eukaryotic cell lines

Policy information about cell lines and Sex and Gender in Research

Cell line source(s)	HEK-293T and IMR-90 cells were obtained from ATCC.
Authentication	Human cell lines were authenticated by DNA (STR) profile performed by Eurofins.
Mycoplasma contamination	All cell lines were routinely tested for mycoplasma and were negative.
Commonly misidentified lines (See ICLAC register)	No commonly misidentified cell lines were used.

Animals and other research organisms

Policy information about studies involving animals; ARRIVE guidelines recommended for reporting animal research, and Sex and Gender in Research

Laboratory animals	S6K1 WT/KO, S6K2 WT/KO mice (8-16 weeks of age) were all in C57BL/6J strain. Mice were bred in heterozygosity to obtain WT and KO littermates. Alb-Cre X S6K1/S6K2, Csf1r-Cre x S6K1/S6K2 mice (8-16 weeks of age) in a C57BL/6J strain were used.
Wild animals	No wild animals were used in the study.
Reporting on sex	Female mice were used for the S6K1 aging study. Male mice were used for the HTVi liver senescence experiments.
Field-collected samples	No field-collected samples were used in the study.
Ethics oversight	Animal experiments were conducted in accordance to the UK Animals (Scientific Procedures) Act 1986 and amended regulations (2012) and approved by the Imperial College´s animal welfare and ethical review body under either 70/8700 or 70/09080. Additional mouse experiments were performed according to German law and with the approval of the Regierungspräsidium Karlsruhe (G139/19).

Note that full information on the approval of the study protocol must also be provided in the manuscript.
