# Supplementary material for: Ribosomal S6 kinase 1 regulates inflammaging via the senescence secretome
Source: Nat Aging. 2024 Aug 29;4(11):1544–61. doi: 10.1038/s43587-024-00695-z (PMC11564105; doi:10.1038/s43587-024-00695-z)
Supplement: Supplementary file 18 — Unprocessed western blots. [file 43587_2024_695_MOESM18_ESM.pdf]

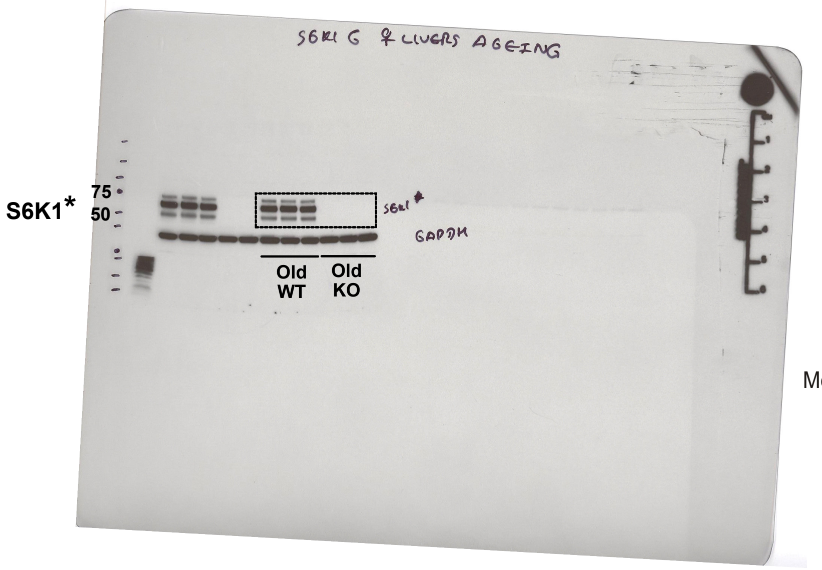

Uncropped images from immunoblotting in **Fig. 1b.**

Black dotted boxes indicate cropped images shown in the indicated Figure Panels.

Molecular weights (kDa) of size markers are shown as indicated.

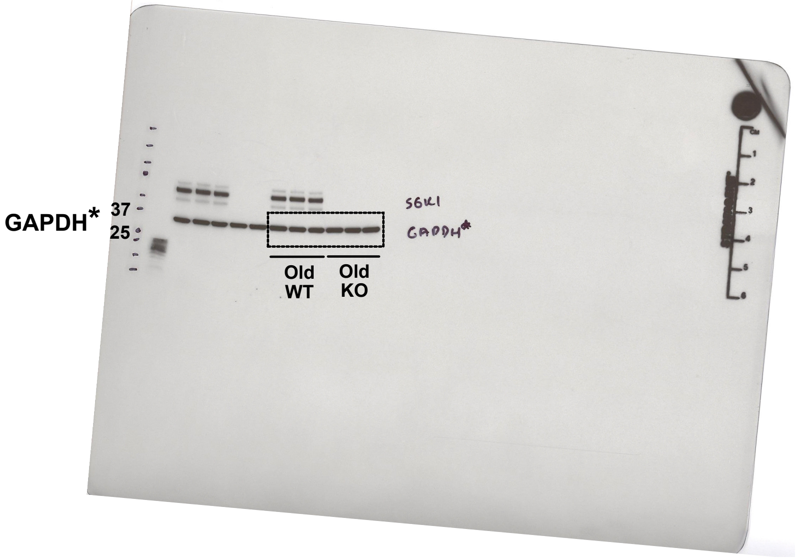

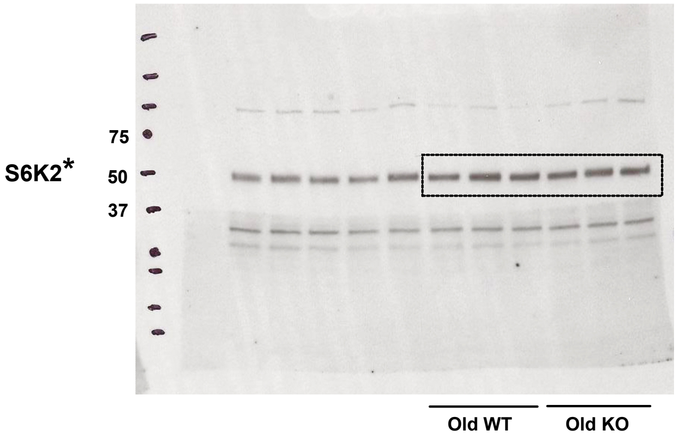

Membrane was cut to simultaneously incubate with multiple antibodies.
