# Supplementary material for: Ribosomal S6 kinase 1 regulates inflammaging via the senescence secretome
Source: Nat Aging. 2024 Aug 29;4(11):1544–61. doi: 10.1038/s43587-024-00695-z (PMC11564105; doi:10.1038/s43587-024-00695-z)
Supplement: Supplementary file 19 — Unprocessed western blots. [file 43587_2024_695_MOESM19_ESM.pdf]

Uncropped images from immunoblotting in **Fig. 5j.**

Black dotted boxes indicate cropped images shown in the indicated Figure Panels.

Molecular weights (kDa) of size markers are shown as indicated.

S6K1*

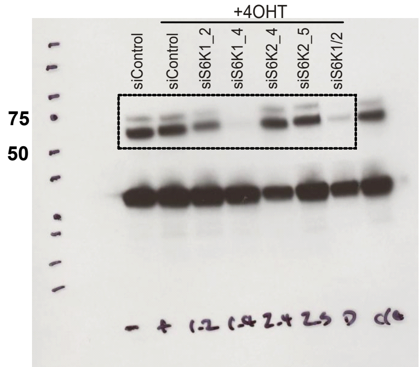

S6K2*

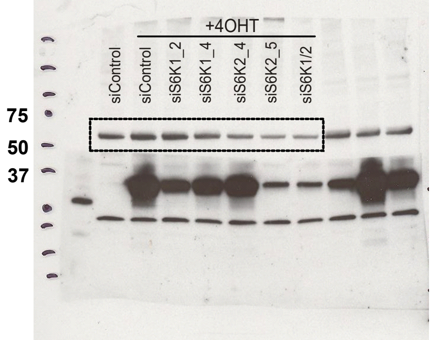

Membrane was cut to simultaneously incubate with multiple antibodies.

IL-1β*

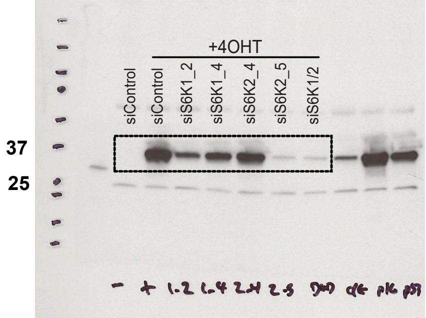

Membrane was cut to simultaneously incubate with multiple antibodies.

GAPDH*

IL-8*

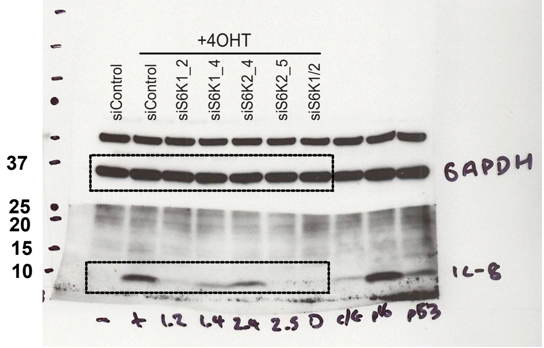

Membrane was cut to simultaneously incubate with multiple antibodies.
