# Supplementary material for: Ribosomal S6 kinase 1 regulates inflammaging via the senescence secretome
Source: Nat Aging. 2024 Aug 29;4(11):1544–61. doi: 10.1038/s43587-024-00695-z (PMC11564105; doi:10.1038/s43587-024-00695-z)

S6K1*

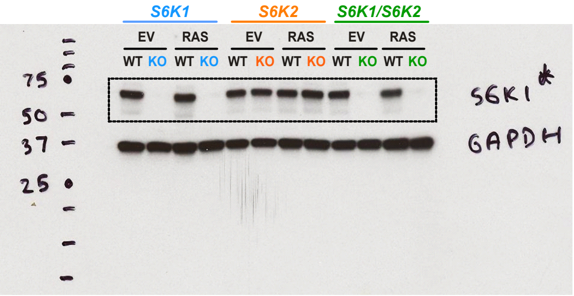

Uncropped images from immunoblotting in **Extended Data Figure 5a.**

Black dotted boxes indicate cropped images shown in the indicated Figure Panels.

Molecular weights (kDa) of size markers are shown as indicated.

GAPDH*

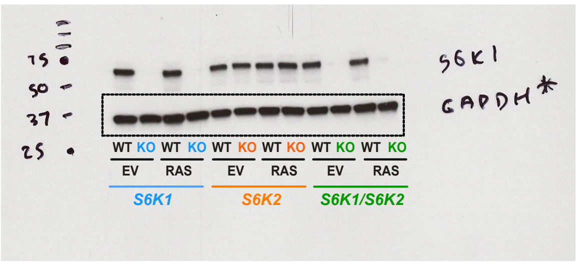

S6K2*

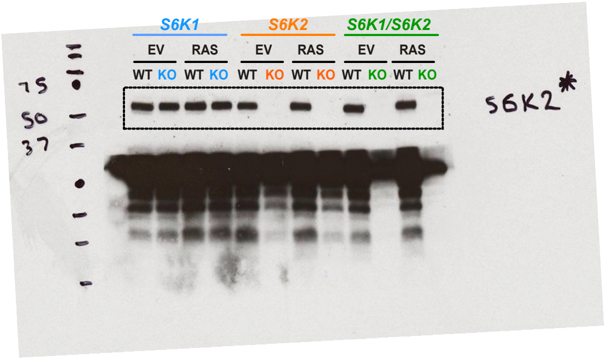

pS6$^{S240/S244}$*

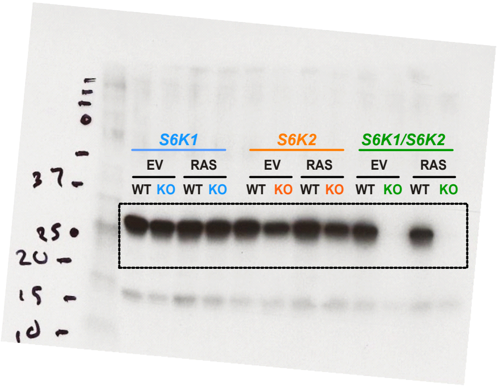

HRAS*

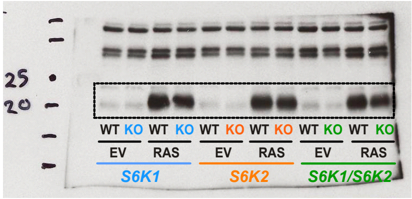

Supplement: Supplementary file 20 — Unprocessed western blots. [file 43587_2024_695_MOESM20_ESM.pdf]
